# Metabolic Reprogramming in Glioma

**DOI:** 10.3389/fcell.2017.00043

**Published:** 2017-04-26

**Authors:** Marie Strickland, Elizabeth A. Stoll

**Affiliations:** Institute of Neuroscience, Newcastle UniversityNewcastle upon Tyne, UK

**Keywords:** glioma, cancer, brain tumors, mitochondria, metabolism, autophagy, catabolism, biosynthesis

## Abstract

Many cancers have long been thought to primarily metabolize glucose for energy production—a phenomenon known as the Warburg Effect, after the classic studies of Otto Warburg in the early twentieth century. Yet cancer cells also utilize other substrates, such as amino acids and fatty acids, to produce raw materials for cellular maintenance and energetic currency to accomplish cellular tasks. The contribution of these substrates is increasingly appreciated in the context of glioma, the most common form of malignant brain tumor. Multiple catabolic pathways are used for energy production within glioma cells, and are linked in many ways to anabolic pathways supporting cellular function. For example: glycolysis both supports energy production and provides carbon skeletons for the synthesis of nucleic acids; meanwhile fatty acids are used both as energetic substrates and as raw materials for lipid membranes. Furthermore, bio-energetic pathways are connected to pro-oncogenic signaling within glioma cells. For example: AMPK signaling links catabolism with cell cycle progression; mTOR signaling contributes to metabolic flexibility and cancer cell survival; the electron transport chain produces ATP and reactive oxygen species (ROS) which act as signaling molecules; Hypoxia Inducible Factors (HIFs) mediate interactions with cells and vasculature within the tumor environment. Mutations in the tumor suppressor p53, and the tricarboxylic acid cycle enzymes Isocitrate Dehydrogenase 1 and 2 have been implicated in oncogenic signaling as well as establishing metabolic phenotypes in genetically-defined subsets of malignant glioma. These pathways critically contribute to tumor biology. The aim of this review is two-fold. Firstly, we present the current state of knowledge regarding the metabolic strategies employed by malignant glioma cells, including aerobic glycolysis; the pentose phosphate pathway; one-carbon metabolism; the tricarboxylic acid cycle, which is central to amino acid metabolism; oxidative phosphorylation; and fatty acid metabolism, which significantly contributes to energy production in glioma cells. Secondly, we highlight processes (including the Randle Effect, AMPK signaling, mTOR activation, etc.) which are understood to link bio-energetic pathways with oncogenic signals, thereby allowing the glioma cell to achieve a pro-malignant state.

## Glioma: an intractable cancer

Glioma is the most common form of adult-onset primary malignant brain tumor, with 5 cases per 100,000 people diagnosed each year (CBTRUS, 2009). In approximately 55% of cases, glioma manifests as a grade IV astrocytoma (called glioblastoma, or GBM), a highly aggressive tumor. Patients with GBM currently receive a dire prognosis, with a median survival of just over one year (Ohgaki and Kleihues, 2005). Patients with lower-grade gliomas, including Grade II-III astrocytoma and oligodendroglioma, have a better prognosis, although these tumors do increase in grade over time, with a median survival rate of approximately five years (Dolecek et al., 2012).

Genetically-defined subtypes of GBM have been identified through large-scale analysis of patient tissue samples (Verhaak et al., 2010). The Classical subtype is characterized by epidermal growth factor receptor (EGFR) tyrosine kinase amplification, p16/p14 deletion, and high levels of Notch protein expression; the Mesenchymal subtype is characterized by Neurofibromatosis 1 (NF1)/Phosphatase and tensin homolog (PTEN) co-deletion, high MET protein expression, and high levels of inflammation; the Pro-neural subtype generally demonstrates high expression of platelet-derived growth factor receptor (PDGFR) A as well as mutations in the tumor suppressor p53 and the Kreb's Cycle enzyme isocitrate dehydrogenase 1 (IDH1); these mutations are also common in low-grade gliomas. The Neural subtype has transcriptional similarities to normal neurons, including expression of neurofilament protein, synaptic proteins, and chloride transporters, despite having a morphologically glial appearance similar to other subtypes of GBM.

Current treatments for malignant glioma include a combination of surgical resection, radiotherapy or radiosurgery, and chemotherapy (typically temozolomide). There is a great need to develop new therapies, to improve overall survival time and quality of life for these patients. Recent efforts in drug development for slowing glioma progression has focused on the inhibition of growth factor receptor tyrosine kinases, cell-surface receptors, and pro-malignancy kinase signaling pathways. Cancer cell metabolism also provides ample scope for the identification of new therapeutic targets. This review aims to comprehensively summarize the current state of knowledge regarding glioma cell metabolism, as well as the open questions in this fast-moving field.

## A note on model systems for studying cancer cell metabolism *in vitro* and *in vivo*

The field of cancer cell metabolism has exploded in the past ten years, but why now? Recently, it has become appreciated that classical model systems for studying malignant glioma may not perfectly reproduce the biochemistry or physiology of human tumors. Two critical factors have greatly impacted the recent boom in this research area: the development of new cell culture techniques (particularly neurospheres and serum-free adherent primary cultures) and new animal models of malignant glioma, including patient-derived xenograft (PDX) models, genetically-engineered mouse (GEM) models, and syngeneic transplant models.

Cell culture is a critical tool in our field, but care must be taken in the process of cell isolation and maintenance. Unfortunately, genetic profiling has undermined confidence in the integrity of older cell lines. For example, the sub-clone of U-87 commonly used in many labs today (U-87-MG) is not actually derived from the original tumor cells, although it probably is derived from a human glioblastoma (Allen et al., 2016). Genetic profiling of the U-251 cell line and its derivatives demonstrated that sub-clones share a common origin from a single human glioblastoma, but have undergone significant genetic drift. U-251 and related cells used today have lost the common GBM characteristics observed in early-passage, including amplification of chromosomes 3, 7, 15, and 17, and loss of chromosomes 10, 13, and 14, although all sub-clones maintain homozygous deletion of the p16/p14ARF locus (Torsvik et al., 2014).

Culturing cancer cells in the presence of serum has been shown to alter their epigenetic and biochemical characteristics, especially leading to deletion of 18q11-23 (Masters et al., 2001), a locus containing several protein-encoding genes that play key roles in lipid metabolism and oxidative phosphorylation (Huret et al., 2013). Perhaps such genetic changes may underlie phenotypic alterations observed upon serum exposure, including altered bio-energetic strategies and adaptation in cellular metabolism. Culturing cells with the growth factors bFGF and EGF, but without fetal bovine serum, is now the standard in the field (Fael Al-Mayhani et al., 2009). Patient-derived cells maintained under serum-free conditions in neurospheres or laminin-attached monolayers have been shown to retain the original characteristics of the human tumors (Lee et al., 2006; Pollard et al., 2009) and have a more oxidative phenotype (Lin et al., 2017).

Animal models which effectively reproduce the key characteristics of human tumors are crucial for studying cancer cell metabolism. Xenografts of human glioma cells that have been implanted into the flanks of animals, rather than orthotopically, are not growing in the brain environment with the characteristic nutrient availability of that tissue (Huszthy et al., 2012). Additionally, the common use of immunocompromised mice to prevent rejection of xenografted human cells, eliminates immune responses which are thought to play roles in driving tumor progression (Budhu et al., 2014). Since the interactions between immune factors, inflammatory responses and metabolic signaling are unknown, it is difficult to judge at this time whether these models are appropriate for investigating these key hallmarks of cancer biology.

Since these cancer models do not reproduce the complex interactions of glioma cells with surrounding brain tissue and immune factors, such systems may not accurately reflect human tumor biology. For this reason, immunocompetent murine models of glioma which are orthotopic and do not utilize serum-exposed cell lines are increasingly considered necessary for testing novel therapeutics prior to clinical investigation in patients (Oh et al., 2014). Bypassing these issues may improve efforts to predict therapeutic response to therapies targeting either cellular bio-energetics or immune factors in animal models.

Moving forward, it would be best for biochemical pathways to be investigated in glioma cells under serum-free conditions *in vitro* and in a relevant biological context *in vivo*. Where possible and relevant in this review, we will note the type of model system used for experimentation.

## Glycolysis and related pathways

### The dogma of glioma cell metabolism: reliance on aerobic glycolysis

Many cancers, including glioma, have long been thought to primarily metabolize glucose for energy production, a phenomenon known as the Warburg Effect, after the first reports of the phenomenon in sarcoma cells by Otto Warburg in 1925 (Warburg, 1925). This process refers to the incomplete, non-oxidative metabolism of glucose even in the presence of oxygen—thought to be characteristic of cancer cells, in comparison to normal cells which readily undergo oxidative phosphorylation (Warburg, 1956).

**Figure 1:**
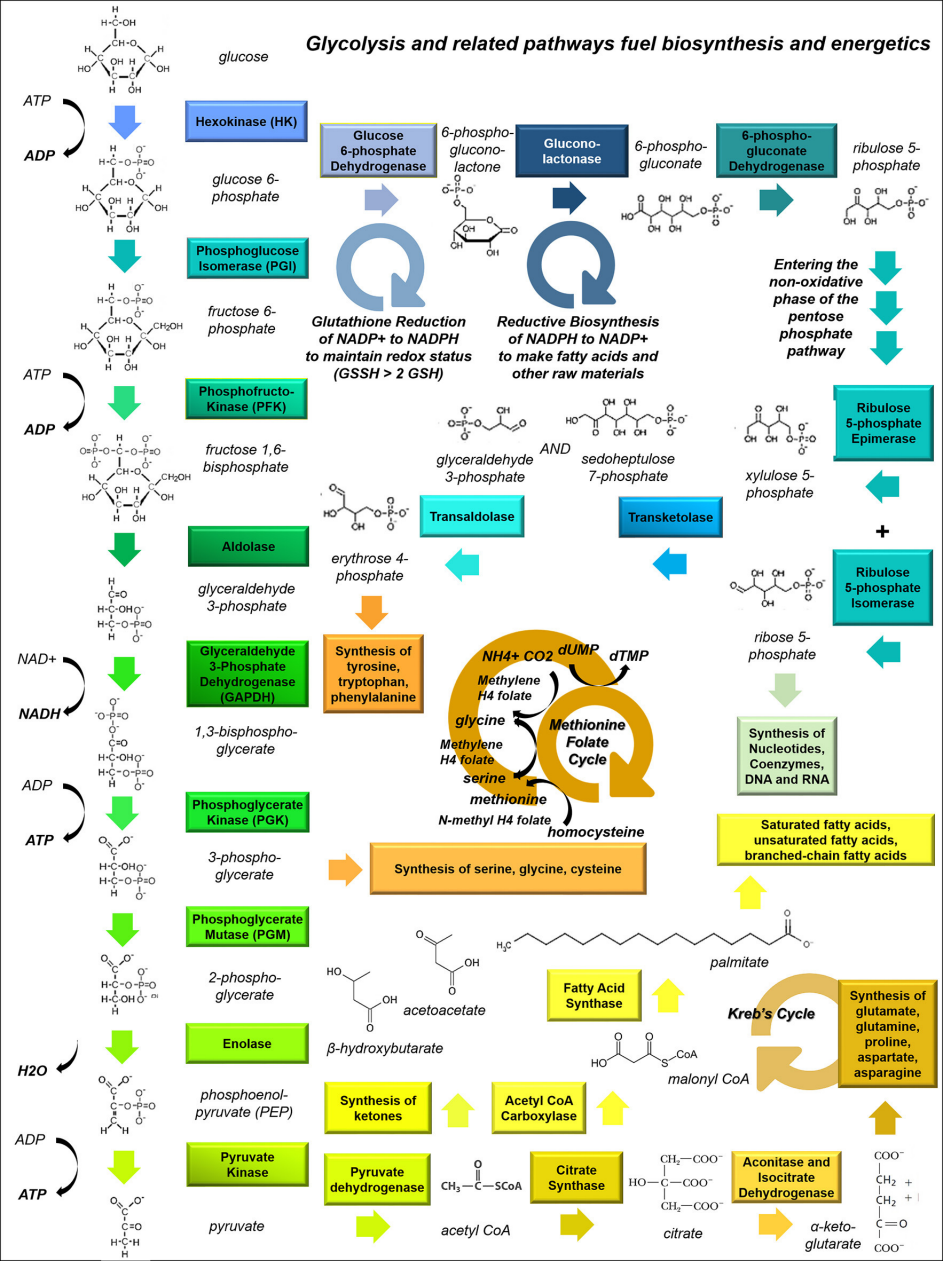
**Glycolysis and related pathways fuel biosynthesis and energy metabolism in cancer cells**. This schematic shows how the glycolytic pathway feeds into other pathways, including the Kreb's Cycle (which fuels amino acid synthesis and provides metabolic intermediates for the electron transport chain), the pentose phosphate pathway (PPP, which is involved in nucleic acid synthesis), and one-carbon metabolism (the folate-methionine cycle).

During oxidative phosphorylation glucose is taken up by respiring cells and undergoes glycolysis; the end-product pyruvate is then able to enter the Kreb's (tricarboxylic acid, or TCA) cycle (Figure 1). This produces NADH (reduced nicotinamide adenine dinucleotide) which is fed into the electron transport chain, yielding 36 molecules of adenosine triphosphate (ATP) per glucose molecule. During aerobic glycolysis however, the end product of glycolysis—pyruvate—is converted into lactate and released into the extracellular space. This process only yields 2 molecules of ATP per glucose molecule, and is inefficient in supporting cellular energy demands (Vander Heiden et al., 2009).

The advantage that the Warburg effect conveys to cancer cells is unclear. It has been posited that ATP levels are not a limiting factor for cancer cells (Vander Heiden et al., 2009), since this metabolic phenotype appears prior to hypoxia (Woolf and Scheck, 2012). It is therefore generally accepted that cancer cells undergo aerobic glycolysis so that the NADH by-product of lactate production can be used to fuel biomass production and lactate can be utilized to acidify the microenvironment, facilitating invasion (Vander Heiden et al., 2009).

While the Warburg Effect has been observed in gliomas and other tumors (Oudard et al., 1996), it has been noted that aerobic glycolysis does not account for the total ATP production in many types of cancer cells—both immortalized cell lines and primary cultures (Vander Heiden et al., 2009)—suggesting that other substrates are being oxidized. When this hypothesis was first formally tested in MCF-7 breast cancer cells in 2002, it was discovered that total ATP turnover was 80% oxidative and 20% glycolytic (Guppy et al., 2002). When this hypothesis was tested in primary-cultured human glioblastoma cells, it was observed that cells were highly oxidative and largely unaffected by treatment with glucose or inhibitors of glycolysis (Lin et al., 2017). Thus, it appears that substrate oxidation can co-exist with aerobic glycolysis and lactate release.

A further complication—alongside intracellular metabolic complexity—is potential heterogeneity in metabolic strategies across different cell types within the tumor. In particular, cancer stem cells which propagate tumor growth and allow recurrence after resection or chemotherapeutic treatment, may exploit different metabolic strategies than other cells within the tumor. For example, a recent study showed that glioma stem cells (GSCs) are less glycolytic than differentiated glioma cells, consuming lower levels of glucose and producing lower amounts of lactate while maintaining higher ATP levels compared with their differentiated progeny. The notorious radio-resistance of this cell population is correlated with higher mitochondrial reserve capacity, leading the authors to conclude that GSCs primarily rely upon oxidative metabolic strategies and will not be vanquished by therapies aiming to inhibit glycolysis (Vlashi et al., 2011).

Imaging studies using radiolabelled ligands also bear out this point in regard to malignant glioma. It has long been appreciated that, from a diagnostic imaging perspective, glucose uptake is not a reliable indicator for malignant gliomas. Thirty five to forty percent of recurrent gliomas in human patients are not observed using positron emission tomography imaging techniques based on fluorodeoxyglucose uptake (e.g., FDG-PET), despite being detected by contrast MRI (Belohlavek et al., 2002). These tumors do not have higher glycolytic rates compared with ongoing brain activity, in line with recent studies suggesting that glucose uptake is insufficient to account for brain tumor metabolism (Maher et al., 2012; Mashimo et al., 2014). Other radiolabelled substrates are now being explored to visualize gliomas, such as the PET tracer 3′-deoxy-3′[(18)F]-fluorothymidine, [(18)F]-FLT. 18F-FLT uptake provides a higher correlation with KI67 index resected tissue compared with 18F-FDG, and has greater predictive power with respect to tumor progression and patient survival (Chen et al., 2005). It may prove useful to consider other metabolic features of gliomas which could be exploited to improve imaging strategies (Albert et al., 2016). Ultimately, while glucose uptake is critically linked to glioma cell metabolism, this process is not sufficient to differentiate gliomas from normal brain or to calculate energy production (Belohlavek et al., 2002; Maher et al., 2012; Mashimo et al., 2014).

### Production of nucleic acids through the pentose phosphate pathway

Glioma cells have particular anabolic needs which can be supplied by glucose uptake and shuttling. A dividing cell must continually produce nucleotides to provide material for DNA replication, RNA transcription, energy currency (e.g., ATP and GTP) and the structural elements of secondary messengers (e.g., cAMP and cGMP). To accomplish this metabolic task, glucose-6-phosphate produced during the initial steps of glycolysis is diverted through the pentose phosphate pathway (PPP), where it is converted to ribose-5-phosphate (Figure 1). With the addition of glutamine, glycine, aspartate, CO_2_ and tetrahydrofolate, ribose-5-phosphate is converted into purine nucleotides, or alternatively combined with bicarbonate, aspartate and glutamine to assemble pyrimidines. Both purines and pyrimidines can also be produced through salvage pathways.

The PPP appears to be particularly active in dividing cells within glioma. Telomerase reverse transcriptase (TERT), which is necessary for maintenance of dividing cells, is associated with increased expression and phospho-activation of PPP enzymes through the generation of reactive oxygen species (ROS) (Ahmad et al., 2016). In contrast, knockdown of TERT causes glycogen accumulation. Interestingly, heterogeneous glioma cells, defined by their rate of cell division, appear to have differential expression of glycolytic and PPP enzymes, suggesting possible metabolic underpinnings for go-versus-grow behavior (Kathagen-Buhmann et al., 2016). Specifically, highly proliferative cells have elevated PPP enzymes and lower expression of glycolytic enzymes, while highly migratory cells have a reverse profile. Thus, it appears that metabolic specialization within tumors prize nucleic acid generation in dividing cell types. Mechanisms controlling this behavior in glioma cells are understudied.

### Transfer of one-carbon units through the folate-methionine cycle

Multiple reactions involving volatile carbons are handled by the paired folate-methionine pathway, including thymidine synthesis and the intraconversion of serine and glycine. Tetrahydrofolate, derived from folic acid, is a versatile carbon donor; it can carry a variety of one-carbon groups including methyls, methylenes, and formyls, making it a highly useful cofactor in biosynthetic reactions (Figure 1). Meanwhile S-adenosylmethionine acts solely as a methyl donor. One-carbon metabolism may play a critical role in cancer metabolism.

One recent study demonstrated that methionine deprivation compromises glioma cell growth (Palanichamy et al., 2016). This metabolic pathway is critical to the process of DNA methylation (Mehrmohamadi et al., 2016), providing a close working connection between cellular metabolism and epigenetic modulation. In other cancer cell types serine supports the production of both S-adenosylmethionine and ATP (Oliver Maddocks et al., 2016), and is required for growth by supporting the folate-methionine cycle (Labuschagne et al., 2014).

Glycine and serine levels increase in cultured rat glioma cells exposed to oxygen and glucose deprivation (Fuchs et al., 2012) and enzymes within this pathway are highly expressed in pseudopalisading cells surrounding necrotic foci (Kim et al., 2015). Compartmentalization of this metabolic activity into specific geographical regions of the tumor—particularly in areas most exposed to toxic compounds released by dying cells—suggests the folate-methionine pathway may play an adaptive role for the growing tumor by accommodating nearby cell death. Currently, this pathway is highly understudied in glioma.

## Kreb's cycle: a central hub for cellular metabolism

**Figure 2:**
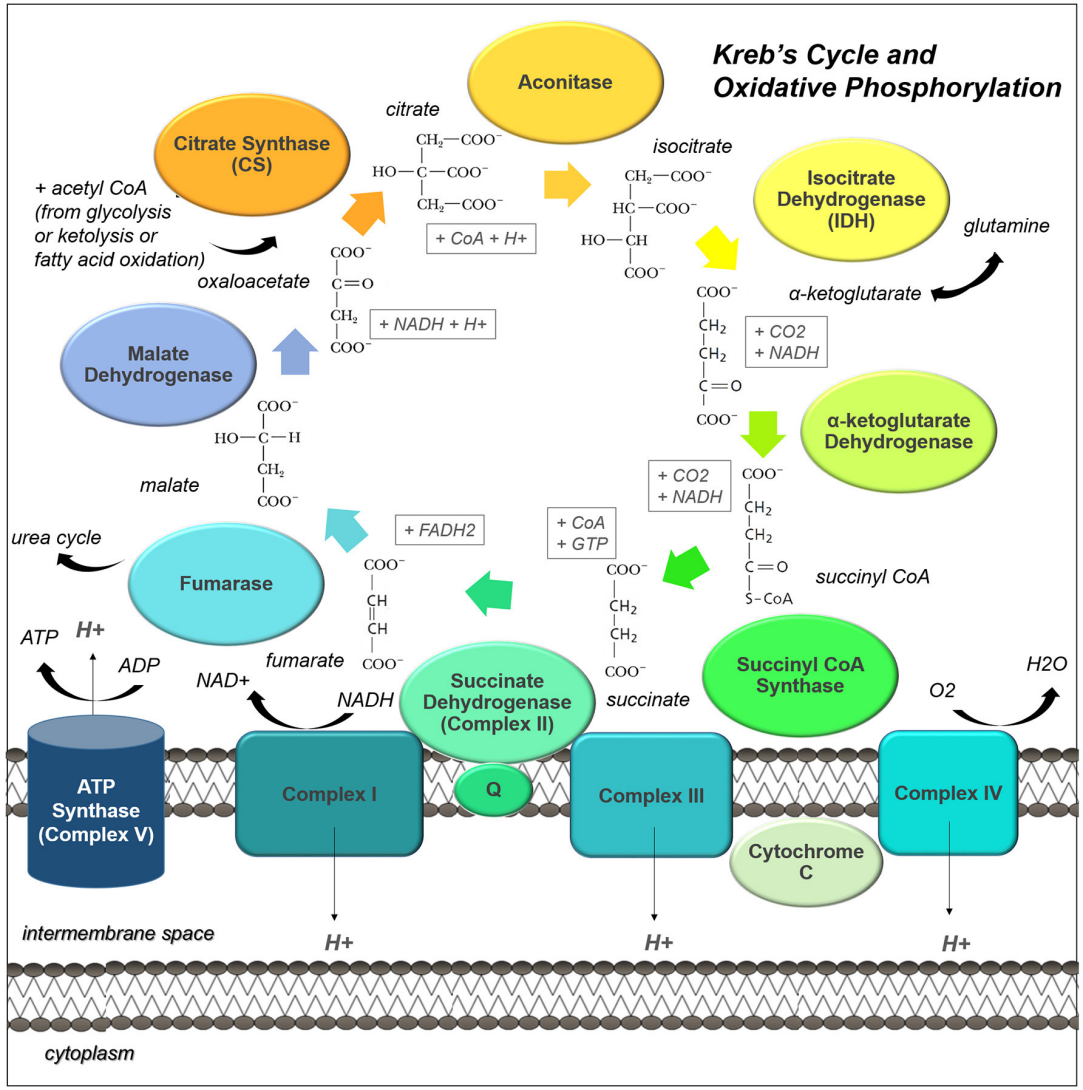
**The Kreb's Cycle and oxidative phosphorylation pathways are central to cell metabolism**. Acetyl CoA from many different sources can enter the Kreb's Cycle (also known as the citric acid cycle, or tricarboxylic acid cycle). This pathway not only drives oxidative phosphorylation by regenerating succinate, but it provides many useful intermediates for biosynthesis. The enzyme complexes of the electron transport chain, which support oxidative phosphorylation, are shown embedded in the inner mitochondrial membrane.

The Kreb's Cycle, is the center of catabolic and anabolic activity within the cell. This process takes place in the mitochondrial matrix and is the main driver of oxidative activity, coupled to both the electron transport chain and many anaplerotic reactions (Figure 2).

The Kreb's cycle can also run in reverse, with citrate being converted to pyruvate by ATP citric lyase and pyruvate being converted to oxaloacetate by pyruvate carboxylase. Malate dehydrogenase and fumarase catalyze their own reverse reactions as well; the resulting fumarate can be used to drive the urea cycle, a coupled reaction used to dispose of excess nitrogen and facilitate protein turnover. Also, gluconeogenesis is initiated from the Kreb's Cycle: phosphoenolpyruvate carboxykinase is a lyase which catalyses the conversion of oxaloacetate to P-enolpyruvate, a gluconeogenic precursor. The Kreb's cycle is therefore a flexible central provider for the catabolic and anabolic needs of the cancer cell.

Importantly, multiple substrates can be used to drive this critical pathway; glycolysis, beta-oxidation, and ketolysis provide acetyl-CoA as an end product. Acetyl-CoA is then converted to citrate by condensation with oxaloacetate (which is regenerated by the completion of the cycle). Some studies using cultured cell lines have suggested that glioma cells use glutamine as a catabolic substrate, entering the Kreb's Cycle through IDH (Yang et al., 2009; Venneti et al., 2015). However, other evidence, using radiolabelled glutamine and glucose *in vivo* to trace the biochemical fate of these substrates in patient-derived xenograft models, suggests the contribution of glutamine to glioma metabolism occurs through hepatic gluconeogenesis and glutamine itself is not metabolized within gliomas (Marin-Valencia et al., 2012). Interestingly, glutamine and glutamate are actually released by glioma cells, affecting the surrounding neural tissues (Buckingham et al., 2011).

Mutations in Kreb's Cycle enzymes are common in cancers. In particular, IDH1 and 2 are present in over 80% of low-grade gliomas and a subset of glioblastomas. IDH1 resides in the cytoplasm, while IDH2 is localized to the mitochondrion; the wild-type enzymatic isoforms catalyze the oxidative decarboxylation of isocitrate to α-ketoglutarate while mutant IDH1 (R132H) and IDH2 (R172K) catalyze the conversion of α-ketoglutarate into the oncometabolite 2-hydroxyglutarate. Interestingly, the evolutionarily-distinct IDH3, which produces NADH not NADPH, does not appear to be mutated at any appreciable rate in glioma cells (Krell et al., 2011). The effects of IDH1 and IDH2 mutations on α-ketoglutarate flux and accumulation of 2-hydroxyglutarate leading to altered intracellular signaling in glioma cells, have been extensively reviewed elsewhere (Waitkus et al., 2015).

Indeed, several metabolic processes are altered in mutant IDH gliomas. Patients with wild-type IDH1 and IDH2 have higher levels of branched-chain amino acids valine, leucine, and isoleucine, and the enzyme that initiates their catabolism (branched-chain amino acid transaminase 1; BCAT1) (Tonjes et al., 2013). When BCAT1 is knocked down with shRNA, glioma cell growth is reduced *in vitro* and *in vivo*, and when treated with gabapentin, a pharmacological inhibitor of BCAT1, glutamate release is also attenuated.

IDH mutations not only affect amino acid metabolism but also lower glucose oxidation through inhibitory phosphorylation of pyruvate dehydrogenase (PDH) (Izquierdo-Garcia et al., 2015). In addition, IDH1 mutant glioma cells show greater flux through pyruvate carboxylase (Izquierdo-Garcia et al., 2014) leading to greater production of oxaloacetate. These results suggest that IDH1 mutant glioma cells adaptively run the Kreb's Cycle backwards, perhaps to produce sufficient succinate to power the electron transport chain. Reverse flux of α-ketoglutarate to acetyl-CoA is a necessary early step in phospholipid synthesis for IDH-wildtype glioma cells and normal astrocytes, especially under hypoxic conditions when hypoxia-inducible factor-1α (HIF1α) is activated (Wise et al., 2011); this lipid production is attenuated in glioma cells with mutant IDH (Chen et al., 2014). IDH1 mutation therefore not only causes 2-hydroxyglutarate build-up, but also broad changes in metabolic strategy.

Other Kreb's Cycle enzymes are mutated, with functional implications, in other tumor types. Mutation or loss of fumarate hydratase (FH) can predispose cells to oncogenic transformation and cyst formation leading to renal cancer and renal cysts, respectively (Adam et al., 2013). Hypermethylation and a reliance on pyruvate carboxylation is observed reliably in paragangliomas with succinate dehydrogenase (SDH) mutations (Lussey-Lepoutre et al., 2015). Mutations in FH and SDH in these cancer cells cause an accumulation of metabolites which leak out of the mitochondrial matrix and inhibit prolyl hydralase (PHD) enzymes, leading to apoptotic resistance and hypoxia signaling (even under oxygen-stable conditions) (King et al., 2006). Interestingly, inhibition of PHDs has been shown to enact hypoxia-related signaling and pro-malignant behavior in glioma cells (Gao et al., 2016). While mutations in FH and SDH have not been observed in glioma, the broad and consistent alterations to metabolic strategies yielded by various alterations in key Kreb's Cycle enzymes across different tumor types suggest that much can be learned about cancer cell metabolism from studies undertaken in other tissues.

The Kreb's Cycle, as a central regulator of anabolic and catabolic metabolism in the cancer cell, is well-placed to coordinate adaptive metabolic strategies. IDH provides a clear example of how a single mutation in this pathway can indicate a unique pathophysiology.

## Functionality of mitochondrial and the mitochondrial electron transport chain in glioma cells

Glioma cells demonstrate alterations to mitochondrial morphology, with some cells containing healthy electron-dense mitochondria and others exhibiting mitochondria with extensive cristolysis; these characteristics are thought to correlate with hypoxia-resistant and hypoxia-sensitive cell types (Arismendi-Morillo and Castellano-Ramirez, 2008).

This finding is compatible with observations of mitochondrial physiology. GSCs have high mitochondrial reserves compared with differentiated cell types; inhibiting neither glycolysis nor oxidative phosphorylation in this cell type has significant effects on energy production (Vlashi et al., 2011). These findings suggest possible mechanisms by which the therapy-resistant GSC may become particularly adaptive from a metabolic standpoint.

Another way to address the question of mitochondrial integrity and functionality is through observations on mitochondrial enzyme expression and activity. Early studies using C6 rat glioma cell xenografts identified lower cytochrome C oxidase (COX, Complex IV) and SDH (Complex II) enzyme expression in more hypoxic areas of the tumor. More recently, one group observed significantly lower Complex II-IV activity in anaplastic astrocytomas and lower Complex I-IV activity in glioblastomas compared with normal brain tissue, using dissociated cells from freshly-frozen human tumors (Feichtinger et al., 2014). Another group, using mass spectrometry to analyse human glioma tissue samples, observed lower expression of some Complex I subunits but higher levels of many oxidative enzymes including catalase (Deighton et al., 2014). Yet another group discovered in a sample of glioma cells a T → C base-pair substitution in the ND6 subunit of Complex I which causes stabilization of the enzyme and resistance to rotenone and hypoxic conditions (DeHaan et al., 2004). A somewhat contrasting picture regarding electron transport capability in glioma cells is emerging from the use of different techniques across different labs.

With the former studies indicating potential dysfunction in electron transport chain Complexes I and IV (which contain mitochondrial-encoded subunits), it is useful to identify whether mitochondrial DNA (mtDNA) itself is intact. Upon disuse in highly glycolytic cells, genetic drift occurs and the mitochondrial genome accumulates mutations, so mtDNA integrity is a good indicator of mitochondrial enzyme function (Greaves et al., 2014). While a number of studies have detected a high number of mtDNA mutations which have risen *de novo* in glioblastomas (Lloyd et al., 2015), a relatively limited fraction of these are predicted or observed to be pathogenic (Vidone et al., 2015). Indeed, experimentally mtDNA-depleted GBM cells grow at a lower rate compared to their parental cells, and take longer to form tumors; moreover, tumors derived from mtDNA-depleted GBM cells recover mtDNA copy number to control levels over the course of tumor formation (Dickinson et al., 2013). These findings suggest that mitochondrial function may be required for glioma initiation or progression.

Overall, these findings are somewhat conflicted regarding a possible impairment of the respiratory chain in glioma, although this issue may potentially be resolved by positing differences in reliance upon oxidative phosphorylation among different cell types within the tumor.

## The contribution of fatty acids to glioma metabolism

### Fatty acid biosynthesis and oxidation

**Figure 3:**
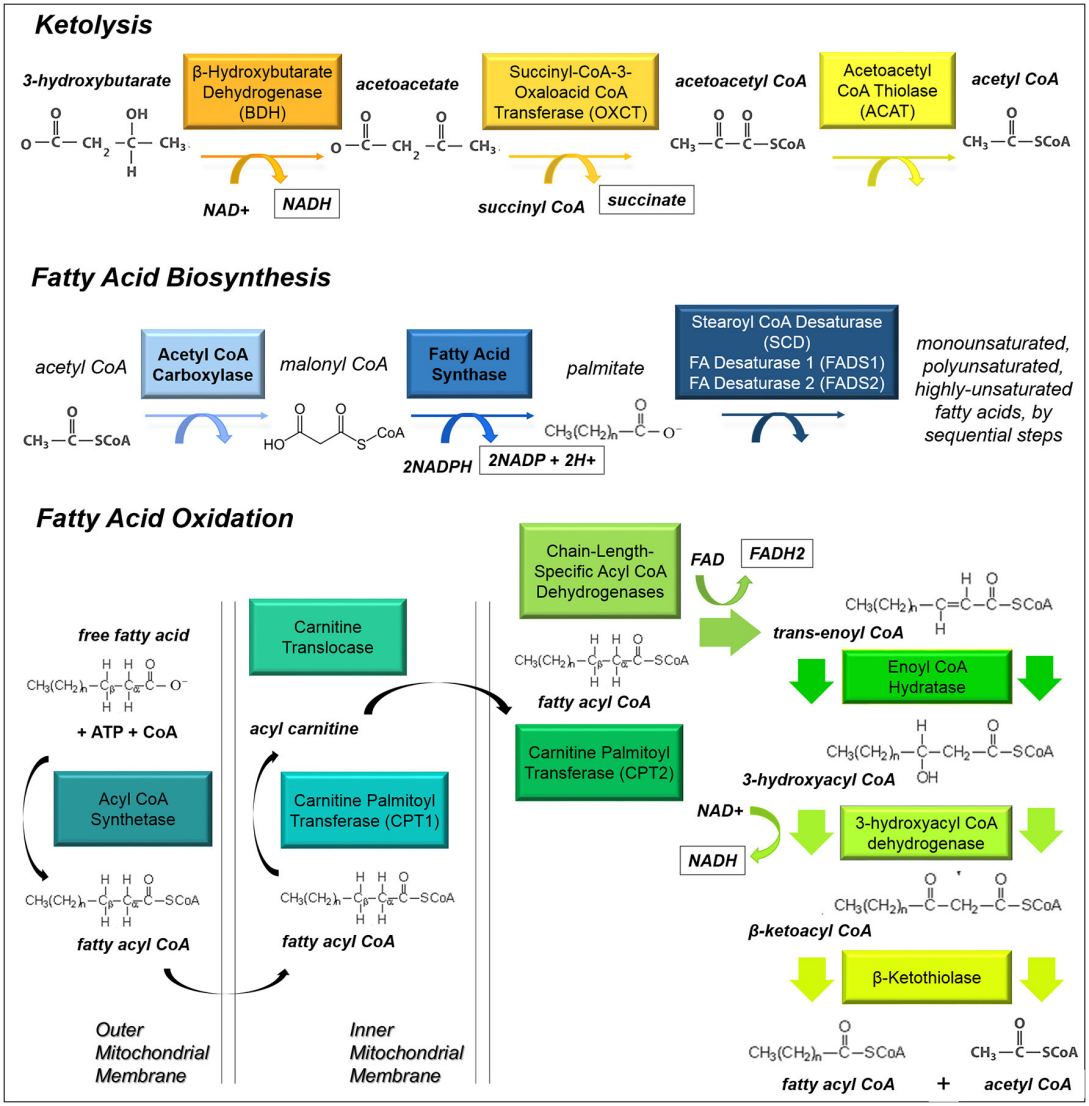
**Ketolysis, fatty acid biosynthesis and fatty acid oxidation**. These pathways provide substrates for glioma cells to make acetyl CoA or utilize it as a building block for lipid-based molecules.

Increasingly, it is appreciated that fatty acids can act as critical bio-energetic substrates within the glioma cell (Figure 3). Recent results from our lab and other groups have demonstrated that glioma cells primarily use fatty acids as a substrate for energy production. Specifically, human glioma cells primary-cultured under serum-free conditions oxidize fatty acids to maintain both respiratory and proliferative activity (Lin et al., 2017). 13C *in vivo* radiolabelling studies conducted in orthotopic mouse models of malignant glioma show that acetate contributes over half of oxidative activity within these tumors, while glucose contributes only a third (Maher et al., 2012; Mashimo et al., 2014).

While glioma cells clearly rely upon fatty acids for energy production, it is not clear whether they acquire fatty acids from the bloodstream or build these carbon chains themselves. Fatty acids pass easily through the plasma membrane, and this may indeed be a nutrient source *in vivo*, but these substrates are not made available in cell culture. However, cells do have access to high concentrations of glucose both *in vitro* and *in vivo*. Glucose can be transported into the cells, converted to fatty acids by the enzyme fatty acid synthase (FASN), then imported into the mitochondria for beta-oxidation (a process known as a Futile Cycle, see next section). Glioma cells contain FASN, and indeed the expression of this enzyme increases with tumor malignancy (Tao et al., 2013). Fatty acid synthesis is initiated from the Kreb's Cycle, where excess citrate is converted to acetyl-CoA by ATP citrate lyase; the product is then carboxylated by acyl-CoA carboxylase 1 (ACC1) to malonyl-CoA, which is then catalyzed by FASN to produce long chain fatty acids, such as palmitate (Currie et al., 2013). Monounsaturated, polyunsaturated, and highly-unsaturated fatty acids are produced by a series of desaturase enzymes: Stearoyl-CoA desaturase and fatty acid desaturases 1 and 2. Fatty acid synthesis has been shown to continue under low-oxygen tension and low-nutrient conditions (Lewis et al., 2015), a process which is activated by HIF1α signaling. Fatty acids are shuttled into lipid droplets upon hypoxia in order to support cell growth and survival upon re-oxygenation (Bensaad et al., 2014).

Inhibition of either fatty acid synthesis or beta-oxidation reduces proliferation of both glioma cells (Grube et al., 2015; Lin et al., 2016) and normal neural stem cells (Lancaster et al., 2013; Stoll et al., 2015). These twinned metabolic pathways provide energy and raw materials for cancer cell growth, and are critically important in glioma cell malignancy. Etomoxir, a specific and irreversible inhibitor of carnitine palmitoyl transferase I (CPT1), the rate-limiting step in beta-oxidation, inhibits respiration and growth of glioma cells, and could provide a new therapeutic option for slowing tumor growth by reducing cellular catabolic activity (Lin et al., 2017). Likewise, orlistat, an inhibitor of FASN which is used in the clinical treatment of obesity, may also hold promise for use as a therapy for malignant glioma (Grube et al., 2014).

There are five main reasons that fatty acids have been overlooked as a metabolic substrate, mostly due to technical considerations involved in cell culture and animal studies. Firstly, fatty acid oxidation is simply an understudied pathway; the role of a metabolic strategy cannot be evaluated if it is not investigated. Secondly, drugs often used to slow glioma through inhibition of glycolysis, such as dichloroacetate (DCA), are non-specific (Michelakis et al., 2010); DCA reduces beta-oxidation as well so cannot be used to parse glycolytic dependency (Bonnet et al., 2007). Thirdly, fatty acids can cause death in cancer cells due to detergent-like effects, especially when not bound to albumin as is commonly the case *in vivo* (Leaver et al., 2002). Fourthly, the reliance of human glioma cells on fatty acid oxidation is abrogated after serum exposure (Lin et al., 2016, 2017), a commonly-used culture method which alters the characteristics of brain-derived cancer cells (Pollard et al., 2009). Studying cells under these conditions may therefore cause an underestimation of oxidative activity. Finally, xenograft tumor models in nude mice, with human glioma cells transplanted into the flank, have often been used to evaluate glycolytic inhibitors but these non-orthotopic transplant models do not have access to brain vasculature and its characteristic nutrient availability (Zhou et al., 2011). Subsequently, it will be useful to study the contribution of beta-oxidation to total energy production in various model systems, across cell types and during tumor progression, and how this process is regulated in glioma.

### Other uses of fatty acids within the cell

Fatty acids synthesized within the cell or obtained from the bloodstream not only contribute to energy production through mitochondrial and peroxisomal beta-oxidation, thereby supplying the Kreb's cycle and electron transport chain. They also play many critical anabolic roles within a cell, forming phospholipids which comprise the plasma membrane and glycerophospholipids which act as signaling molecules. Fatty acids generate paracrine signaling molecules such as endocannabinoids and eicosanoids, driving synthesis of cholesterol and other steroid hormones through the mevalonate pathway, which is highly active in glioblastoma cells (Kambach et al., 2017), and acting as cofactors for fatty acid binding proteins (FABPs), which are necessary for lipid droplet formation under hypoxic conditions (Bensaad et al., 2014). In addition, fatty acids can facilitate post-translational modifications (e.g., palmitoylation) of pro-malignancy proteins. Fatty acids thus play diverse and important roles in the function of cancer cells.

### Ketolysis

Ketolysis is the process by which ketone bodies are broken down to produce acetyl-CoA (Fukao et al., 1997). Often this process occurs in brain tissues when blood sugar levels are low. It has been established that cells within the brain, which normally rely on glucose, can readily switch to oxidizing ketones produced by the liver under necessary circumstances. It is not clear whether ketones might be utilized within glioma cells. Recently radiolabelled acetate has been shown to be taken up as a metabolic substrate for glioma cells (Mashimo et al., 2014). Since ketones can provide necessary substrates for the biosynthesis of fatty acids and can be converted into acetyl-CoA which directly enters the Kreb's Cycle (Fukao et al., 1997), these substrates may provide fuel for a developing tumor (Figure 3).

However, a ketogenic diet has been proposed as a potential therapy for glioma to slow tumor growth and reduce seizure frequency. It is thought that low blood sugar resulting from this dietary intervention prevents glioma cells accessing their preferred fuel source, glucose, and the efficacy of this dietary intervention has been demonstrated in xenograft-transplant models of malignant glioma (Stafford et al., 2010; Abdelwahab et al., 2012; Woolf et al., 2015; Lussier et al., 2016), as well as mouse models of neuroblastoma, a pediatric brain tumor (Morscher et al., 2015). It remains to be seen whether this finding is replicated in human patients; four clinical trials are currently ongoing to evaluate the effects of this diet on overall survival time and quality of life measures in human patients:

- Calorie-restricted, Ketogenic Diet and Transient Fasting During Reirradiation for Patients With Recurrent Glioblastoma (ERGO2), sponsored by the Johann Wolfgang Goethe University Hospital in collaboration with TAVARLIN.
- Pilot Study of a Metabolic Nutritional Therapy for the Management of Primary Brain Tumors (Ketones), sponsored by Michigan State University in collaboration with Sparrow Health System.
- Ketogenic Diet as Adjunctive Treatment in Refractory/End-stage Glioblastoma Multiforme: a Pilot Study, sponsored by Mid-Atlantic Epilepsy and Sleep Center in collaboration with University of Pittsburgh.
- Ketogenic Diet With Radiation and Chemotherapy for Newly Diagnosed Glioblastoma, sponsored by St. Joseph's Hospital and Medical Center Phoenix.

## Functional links between metabolic pathways

**Figure 4:**
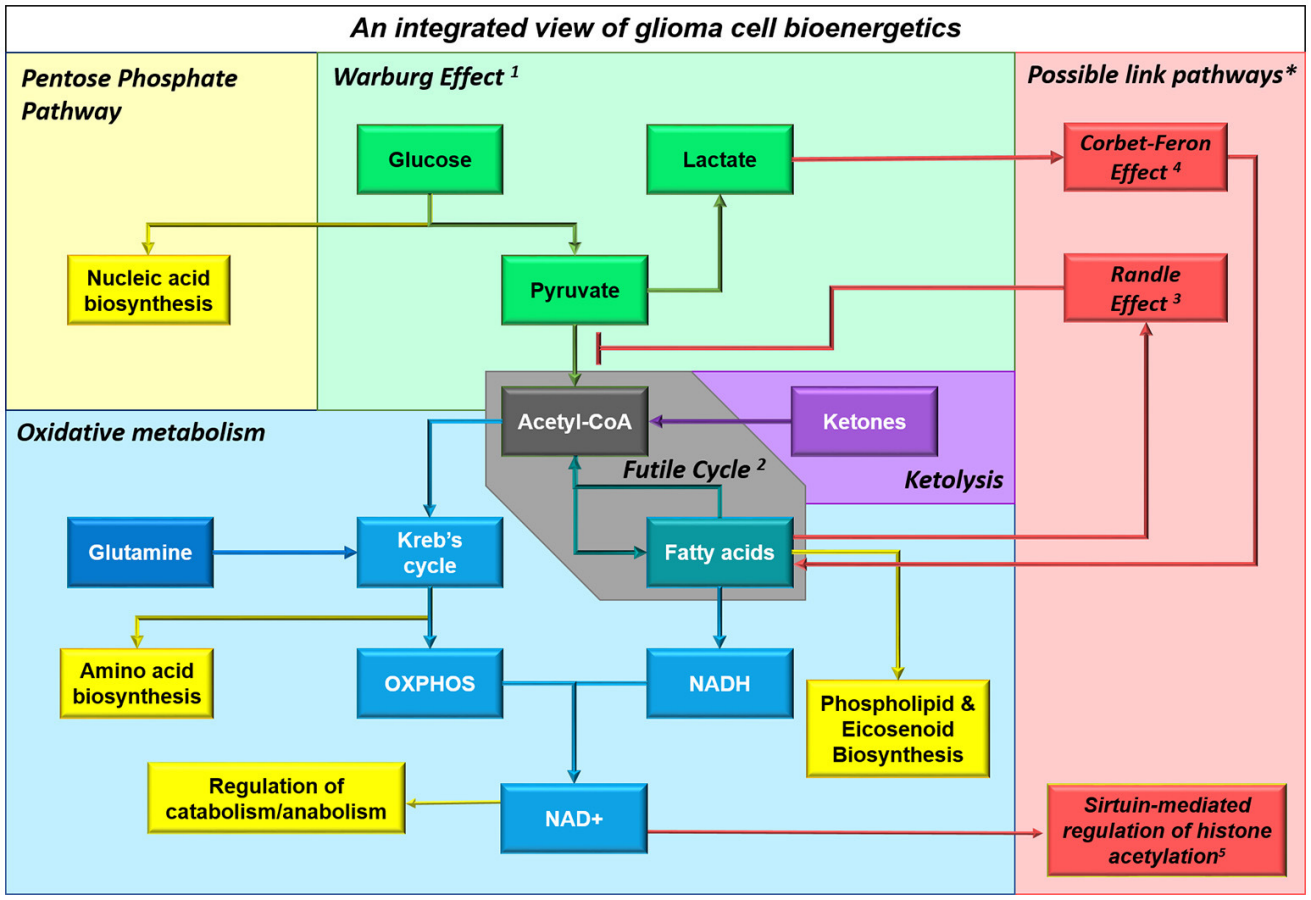
**Multiple substrates can contribute to cellular bioenergetics by providing acetyl CoA**. This schematic shows an integrated view of glioma cell metabolism, demonstrating that ketones providing acetyl CoA will fuel both energy production and biosynthesis of raw materials (e.g., nucleic acids, amino acids, phospholipids and other molecules). The non-oxidative use of glucose by glioma cells is shown in **green**; particularly the ***Warburg Effect***^*1*^ where cancer cells undergo glycolysis that is not followed by oxidation, instead converting the end product pyruvate into lactate and releasing it into the extracellular space. Aerobic respiratory pathways used by glioma cells are shown in **blue**; glioma cells appear to engage in a ***Futile Cycle***^*2*^ (**gray**) where fatty acids are continuously synthesized and oxidized in glioma cells (it has been shown that inhibiting either pathway reduces cellular proliferation and slows tumor progression). Ketolysis, shown in **violet**, and fatty acid oxidation, shown in **teal**, provides acetyl CoA which fuels the Kreb's Cycle and oxidative phosphorylation; acetyl CoA produced from any source facilitates anapleurosis and biosynthesis of many molecules critical to cellular function. Metabolic intermediates, such as those produced by the Kreb's Cycle and oxidative phosphorylation, impinge on other cellular functions, as shown in **yellow**. ^*^Interactions between enzymes that link catabolic pathways, which have been identified in other cancers but not yet studied in glioma, are shown in **red**. These include: the ***Randle Effect***^*3*^ where NADH and acetyl CoA produced during fatty acid (FA) oxidation inhibit pyruvate dehydrogenase, thus promoting glucose's alternative fate (release as lactate), the ***Corbet-Feron Effect***^*4*^ where lactate-induced acidification of the microenvironment promotes the FA oxidation phenotype via acetylation-mediated activation of mitochondrial proteins, and ***sirtuin-mediated regulation of histone acetylation***^*5*^.

It is important to note that catabolic pathways do not exist in isolation; they are inextricably linked to the biology of the cancer cell (Figure 4). Several pathways in particular, which have been identified in other cancer types, may play a role in glioma as well.

### The randle effect: coupling of beta-oxidation and glycolysis?

Bio-energetic pathways in glioma cells do not occur in isolation; importantly, they are connected to each other through cross-signaling. A case in point is the Randle Effect, a prime example of tightly-coordinated cellular energy metabolism which provides a mechanistic link between beta-oxidation and aerobic glycolysis.

In the 1960s and 1970s, Randle showed that NADH and acetyl-CoA produced during beta-oxidation both inhibit the activity of pyruvate dehydrogenase (PDH), thereby promoting the conversion of pyruvate to lactate. Well-characterized in diabetes, the work of Randle and his colleagues reveals that non-oxidative glycolysis can occur alongside the oxidation of other substrates, particularly fatty acids (Randle et al., 1963).

The activity of the PDH complex, which allows the end-product of glycolysis to enter the Kreb's Cycle, is modulated by reversible phosphorylation by PDH kinase (PDK); NADH and ATP produced in the course of beta-oxidation lead to activation of PDK, which in turn phospho-inactivates PDC, leading to lower rates of glucose oxidation and higher rates of lactate release (Holness and Sugden, 2003). In this way beta-oxidation is compatible with ongoing aerobic glycolysis, and in fact could promote the Warburg Effect.

### The corbet-feron effect: a causal link between acidification and beta-oxidation?

A more recent study has established the Corbet-Feron Effect, where lactate-induced acidification of the microenvironment over a period of weeks leads to adaptation of the cancer cell population, promoting beta-oxidation as a metabolic strategy (Corbet et al., 2016). This shift is associated with histone deacetylation in the nucleus and DNA hyper-acetylation in the mitochondria, and is dependent upon acetylation-mediated activation of mitochondrial proteins. This effect was demonstrated in SiHa cervix cancer cells, FaDu pharynx squamous cell carcinoma cells, HCT-116 and HT-29 colon cancer cells, but was not investigated in glioblastoma cells, so it remains to be seen whether this effect is relevant in malignant brain tumors.

### A “futile cycle” can regulate an anabolic-catabolic switch

A futile cycle occurs when a cell runs two identical metabolic pathways simultaneously in opposite directions, with no effect but a small net consumption of ATP. Such processes (e.g., fatty acid biosynthesis and beta-oxidation, which both occur in the glioma cell) may seem paradoxical. Yet futile cycles accomplish two important tasks: Firstly, they ensure the continuous availability of raw materials through biosynthesis (e.g., heavily used molecules such as phospholipids), while providing a good source of ATP and NADH through the catabolism of any excess material; and secondly, they are thought to play a critical role in regulating metabolic processes, as a futile cycle can induce a bi-stable oscillatory state which is highly sensitive to small changes in enzymatic activity and can be used to communicate changes within the cell (Samoilov et al., 2005). Several of the signaling pathways discussed below exert their effects through this mechanism.

## AMP-activated protein kinase: a coordinator of energy metabolism and cell cycle progression

### Structure and function of AMPK

**Figure 5:**
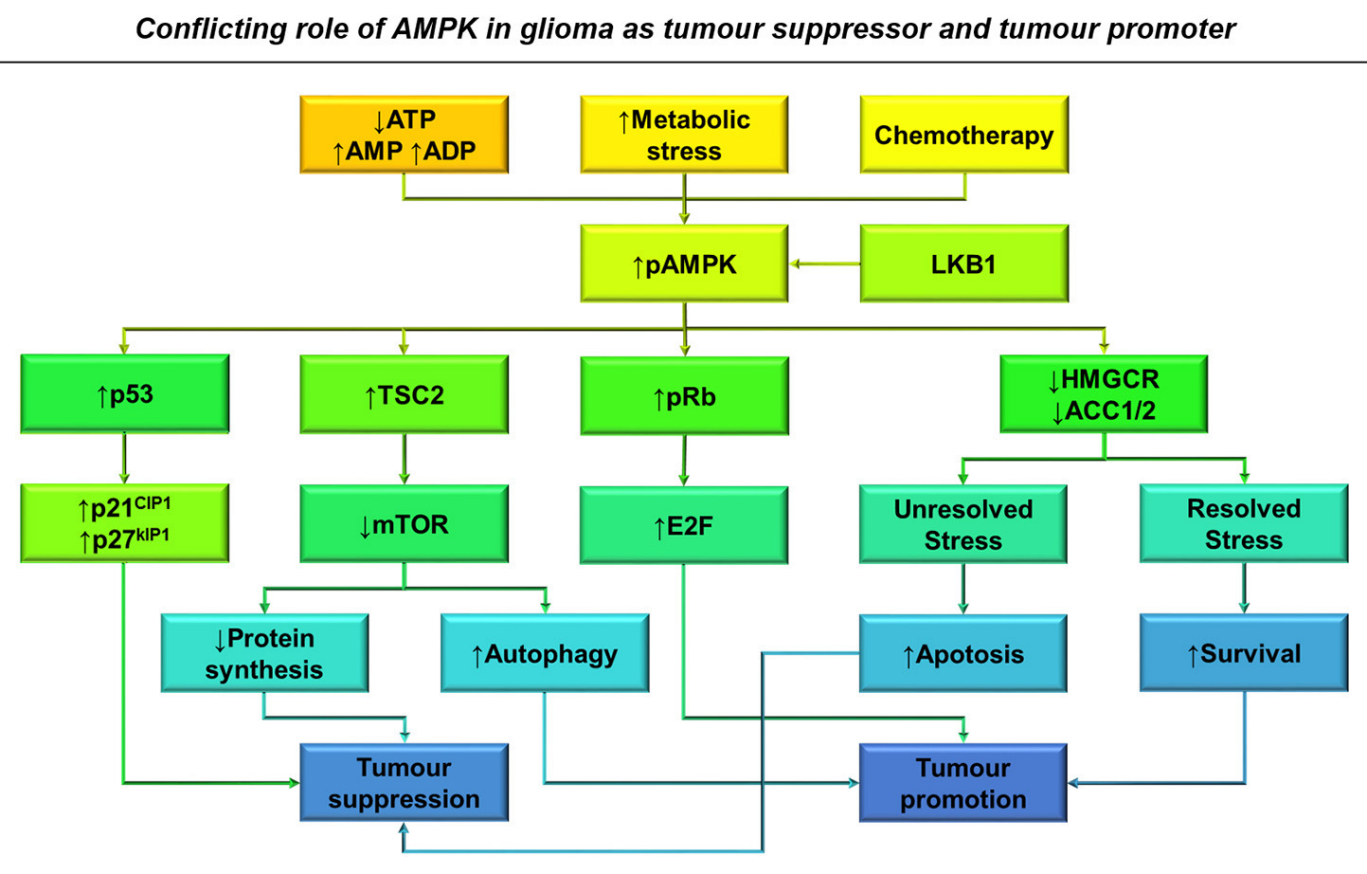
**The conflicting role of AMPK in glioma as a tumor suppressor and tumor promoter**. Increased AMP: ATP and ADP: ATP ratios, metabolic stress and treatment with chemotherapy results in AMPK activation in glioma cells (Hardie and Alessi, 2013). This results in the activation of several downstream pathways that result in both tumor suppression and growth depending on the context of AMPK activation. Primarily AMPK activates p53 transcriptional activity leading to classical cell cycle inhibition, reducing tumor growth through p21^CIP1^ and p27^KIP1^ activation (Jones et al., 2005). AMPK activation also downregulates mTOR signaling resulting in decreased protein synthesis and G2 block, whilst also releasing its constraint on autophagy, increasing glioma survival during chemotherapy (Vucicevic et al., 2009; Misirkic et al., 2012). Unresolved stress over prolonged periods can also result in increased apoptosis and therefore decreased viability. By increasing Rb phosphorylation, AMPK activation is also able to overcome cell cycle inhibition (Ríos et al., 2014).

AMP-activated protein kinase (AMPK) is the central energy sensor within all mammalian cells and consists of three subunits: the catalytic α-subunit and two regulatory β- and γ-subunits (Hardie and Alessi, 2013). AMPK senses increased ADP and AMP during periods of energy stress, regulating the switch from anabolism to catabolism in order to regain cellular energy homeostasis (Figure 5).

The γ-subunit of AMPK contains 4 cystathione β-synthase (CBS) motifs, which have the ability to bind ATP, AMP and ADP in accordance with their cellular ratios (Hardie and Alessi, 2013). AMP is constitutively bound to the 4th CBS motif whilst the 2nd remains empty, allowing the remaining two motifs to bind ATP, AMP, and ADP. When ATP levels are high AMPK remains inactive, however when cellular energy levels are low, AMP and ADP compete for binding to the γ-subunit (Hardie et al., 2012). Binding of AMP and ADP changes the morphology of AMPK, requiring N-terminal myristylation of the β-subunit, and promoting phosphorylation of AMPK-α at Thr172 (Oakhill et al., 2009).

Liver kinase B1 (LKB1) is the major activator of AMPK and constitutively phosphorylates Thr172, however high ATP binding promotes its rapid dephosphorylation (Hardie and Alessi, 2013). The conformational change in AMPK upon ADP/AMP binding inhibits this dephosphorylation increasing AMPK activation 100-fold (Hardie et al., 2012). AMP itself has the ability to cause further allosteric activation of AMPK another 10-fold, and in this manner AMPK can respond to a variety of changes in cellular energy levels and augment its response accordingly (Hardie et al., 2012).

The two main substrates of AMPK are ACC and hydroxylmethylglutaryl-CoA reductase (HMGCR), which regulate fatty acid and cholesterol metabolism respectively (Hardie and Pan, 2002). When activated, AMPK inhibits ATP-consuming anabolic processes by phosphorylating ACC and HMGCR. Through inhibiting both ACC1 and ACC2, AMPK is able to inhibit the production of malonyl-CoA, a precursor for fatty acid synthesis and inhibitor of CPT1, thus alleviating the rate-limiting step of beta-oxidation (Hardie and Pan, 2002). Additionally, AMPK activation also plays a role in other metabolic pathways, by inhibiting mechanistic target of rapamycin (mTOR) and activating Raptor and tumor suppressor complex (TSC1/2) to inhibit protein and mRNA translation and increase glycolysis by inhibiting rate-limiting enzymes (Hardie and Pan, 2002; Hardie, 2011; Hardie et al., 2012; Hardie and Alessi, 2013). Through these mechanisms, AMPK is able to overcome ATP depletion and energy stress maintaining cellular energy homeostasis.

### Role of AMPK in cancer

The role of AMPK as both a tumor suppressor and potential oncogene has been well debated; it appears that the role of AMPK is often dependent on metabolic status of the cell and phosphorylation status of the protein itself. AMPK inhibits cell cycle progression whilst also providing protection from metabolic stress induced by chemotherapeutic agents in glioma, fibrosarcoma, and melanoma cell lines (Vucicevic et al., 2011). Both overexpression and ablation of AMPK can induce cell cycle dysfunction, suggesting that AMPK has a role in both tumor suppression and progression within transformed cells (Max Banko et al., 2011). AMPK integrates inputs from oncogenic signaling and energy metabolism before committing the cell to undergo division (Mukherjee et al., 2008), and its control is further complicated by mutations in tumor suppressor and metabolic pathways.

### Role of AMPK as a tumor suppressor in glioma

Cell cycle inhibition resulting from AMPK activation can occur at both the G1/S and G2/M phases of the cell cycle by differing mechanisms (Guo et al., 2010, Figure 5). AMPK has the ability to bind and phosphorylate p53 and activate TSC2, in order to halt cell cycle progression until homeostasis is restored (Jones et al., 2005; Vucicevic et al., 2011). AMPK also activates p21^CIP1^ and p27^KIP1^, both directly and indirectly through p53. Both these proteins act as cyclin-dependent kinase inhibitors and prevent Rb from releasing E2F thus preventing entry into the cell cycle (Isakovic et al., 2007). By inhibiting fatty acid synthesis, AMPK is able to inhibit G2/M phase progression through regulating the biosynthesis of membrane components required for cytokinesis (Guo et al., 2010).

Within glioma, AMPK activation has been shown to promote apoptosis, better prognosis, and increased response to chemotherapy depending on metabolic context (Isakovic et al., 2007; Zadra et al., 2015). Glucose withdrawal causes more AMPK phosphorylation and apoptosis in astrocytoma cells compared with normal astrocytes due to inefficient mTOR signaling (Mukherjee et al., 2008). Additionally, in low density cultures of U-251 cells, metformin-mediated AMPK activation inhibits proliferation at G0/G1 phase, while in substrate-limiting high-density cultures metformin promotes apoptosis; interestingly, primary rat astrocytes are resistant to the effects of metformin (Isakovic et al., 2007). This serves as a prime example of differential AMPK signaling due to substrate availability and cellular status (Figure 5).

Through tightly regulating mTOR, AMPK moderates the progression, prognosis and resistance of malignant gliomas (Aldea et al., 2011; Vucicevic et al., 2011). Within serum-free primary-cultured human GSCs, metformin-mediated activation of AMPK and FOXO3 induces differentiation and reduces tumourigenic capacity *in vivo* (Sato et al., 2012). However, this effect was only observed during culture at non-physiological glucose concentrations (≥17.5 mM). Resistance to therapy is a major hurdle in overcoming brain tumor recurrence but it has been shown that AMPK activation can enhance glioma response to temozolomide (Zhang et al., 2010). Furthermore, metformin treatment enhances the effects of temozolomide *in vitro* and *in vivo*, correlating with improved therapeutic response in patients (Aldea et al., 2011; Sesen et al., 2015).

### Role of AMPK in glioma progression

Oncogenic events often result in AMPK activation which, if AMPK acted solely as a tumor suppressor, would be highly counter-productive for tumor growth (Ríos et al., 2014). AMPK activity correlates with increased proliferation in clinical samples, U-87-MG cells, and mouse astrocytoma models, mediating increased Rb phosphorylation and cell cycling *in vitro* (Rios et al., 2013, Figure 5). Radio- and temozolomide- resistant human GSC clones show upregulation of genes associated with autophagy and lipid catabolism alongside increased AMPK phosphorylation (Ye et al., 2013). Additionally, AMPK has also been shown to play a role in increased glioma cell migration and survival in response to glucose withdrawal within the U-251-MG human cell line (Godlewski et al., 2010).

AMPK is also able to induce autophagy by downregulating mTOR activity, inducing the recycling of cellular components and the production of ATP during starvation. By inhibiting cholesterol catabolism in U-251 cells, autophagy was upregulated in an AMPK-dependent manner to protect against apoptosis (Vucicevic et al., 2009). By inducing autophagy and inhibiting caspase-3 and p53 mediated apoptosis, AMPK activation can increase glioma cell viability and has helped establish a role for AMPK in tumor growth and decreased patient survival (Rios et al., 2013; Liu et al., 2014, Figure 5).

### AMPK as a putative futile cycle regulator

**Figure 6:**
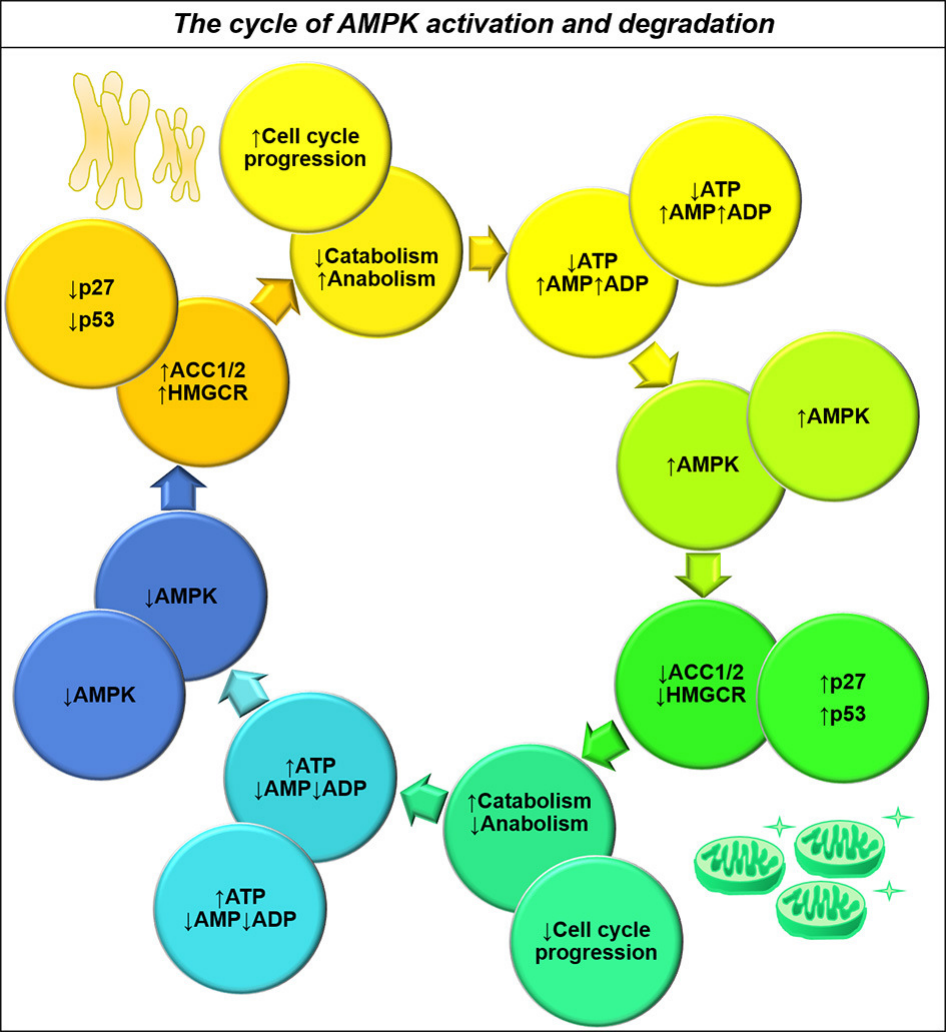
**The dual role of AMPK activity in maintaining energy homeostasis and ensuring nutrient sufficiency for cell cycle progression**. Upon activation by decreased ATP availability, AMPK acts to inhibit energy consuming pathways such as cholesterol and fatty acid synthesis (Hardie and Pan, 2002), whilst also increasing the activity of cell cycle inhibitors p27 and p53 (Isakovic et al., 2007). This tumor suppression acts to restore energy homeostasis by inhibiting cell cycle progression, resulting in decreased AMPK activity and a switch from catabolism to anabolism for completion of the cell cycle. AMPK thereby acts to maintain energy homeostasis and ensure nutrient sufficiency for cell cycle progression.

The contradictory roles of AMPK may be resolved by considering how a cycling cell achieves metabolic homeostasis. DNA replication and cytokinesis undertaken during the process of cell division require much energy, yet the mechanisms by which glioma cells couple catabolic activity to cell cycle progression are not well understood. AMPK and its family members, as nutrient-sensing effector proteins, are well-placed to act as regulators of a futile cycle to accomplish this task. An increased AMP/ATP ratio activates AMPK, which inhibits biosynthetic processes and activates beta-oxidation by inhibiting ACC (Hardie and Pan, 2002; Hardie et al., 2012). AMPK simultaneously acts to inhibit cell cycle progression by activating tumor suppressor proteins (Jones et al., 2005; Liang et al., 2007). Once the cell has enough energy, AMPK is no longer activated and the activated protein is degraded, allowing release from the cell cycle checkpoint. The process of mitosis then presumably depletes energy stores allowing AMPK to be activated again (Figure 6). Interestingly, knockdown and overexpression of this kinase cause the same effect in cells: halting of the cell cycle resulting in aneuploidy (Max Banko et al., 2011). A carefully-regulated, cyclical pattern of AMPK activity—and functional downstream effector molecules—may be required for cycling cancer cells to function properly.

## Mammalian target-of-rapamycin: a key to glioma cell resiliency

### Structure and function of mTOR

mTOR integrates signaling from growth factor pathways with cellular energy and nutrient levels, co-ordinating this activity with biosynthetic machinery and cell cycle machinery (Sarbassov and Sabatini, 2005; Duzgun et al., 2016). Control of protein synthesis and cell cycle entry by mTOR is mediated through the mTOR Complex 1 (mTORC1) and the adaptor protein Raptor, while metabolic effects of mTOR occur through association with the mTORC2 complex with the adaptor-protein Rictor (Akhavan et al., 2010; Masui et al., 2013, Figure 7).

**Figure 7:**
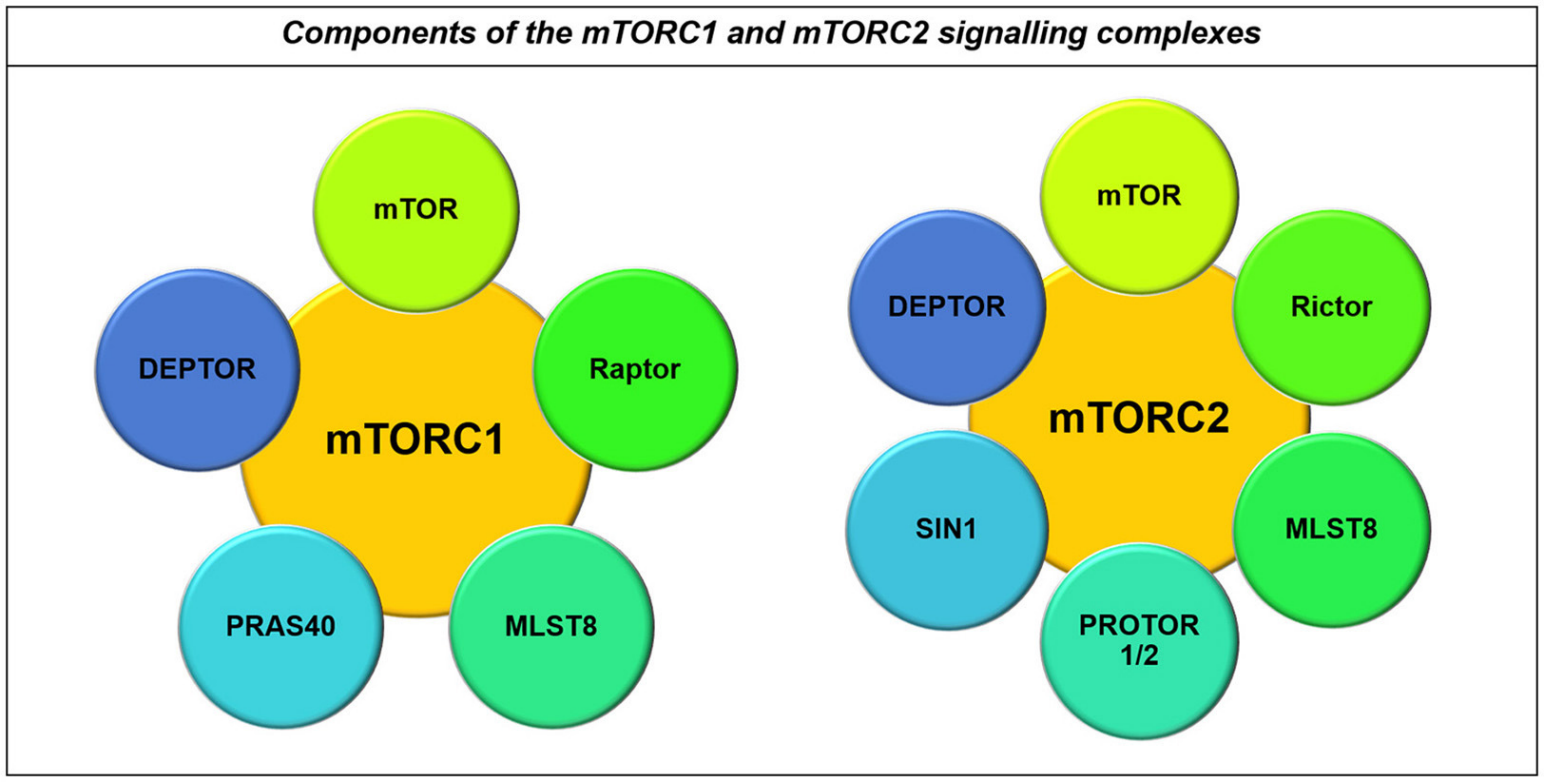
**Components of the mTORC1 and mTORC2 signaling complexes**. Both the mTORC1 and mTORC2 complexes contain mTOR, MLST8, and DEPTOR. However, their major differences lie in the co-binding of Raptor to the mTORC1 complex and Rictor to the mTORC2 complex, in addition to their other binding partners including PRAS40 and PROTOR1/2 and SIN1 respectively (Laplante and Sabatini, 2012).

mTOR acts downstream of many tumor suppressor and oncogenic pathways, which tightly regulate its activity under normal circumstances. However, loss of tumor suppressors such as p53 or oncogenic activity which converges on PI3K (phosphatidylinositol 3-kinase) can augment mTOR activity, resulting in glioma development (Cancer Genome Atlas Research Network, 2008; Akhavan et al., 2010; Levine and Puzio-Kuter, 2010; Duzgun et al., 2016).

### mTOR controls protein synthesis through mTORC1

The TSC1/2 complex is the major upstream regulator of mTOR through inactivating Ras-homolog enriched in brain (RHEB) GTPase activity. Upon mitogenic signaling the TSC1/2 complex is stabilized, allowing RHEB to freely activate mTORC1 (Duzgun et al., 2016). Active mTORC1 then phosphorylates S6K1 at Thr389 and 4E-BP1 at Ser65 to initiate protein translation and ribosome biogenesis, respectively (Dennis et al., 2001, Figure 8A).

**Figure 8:**
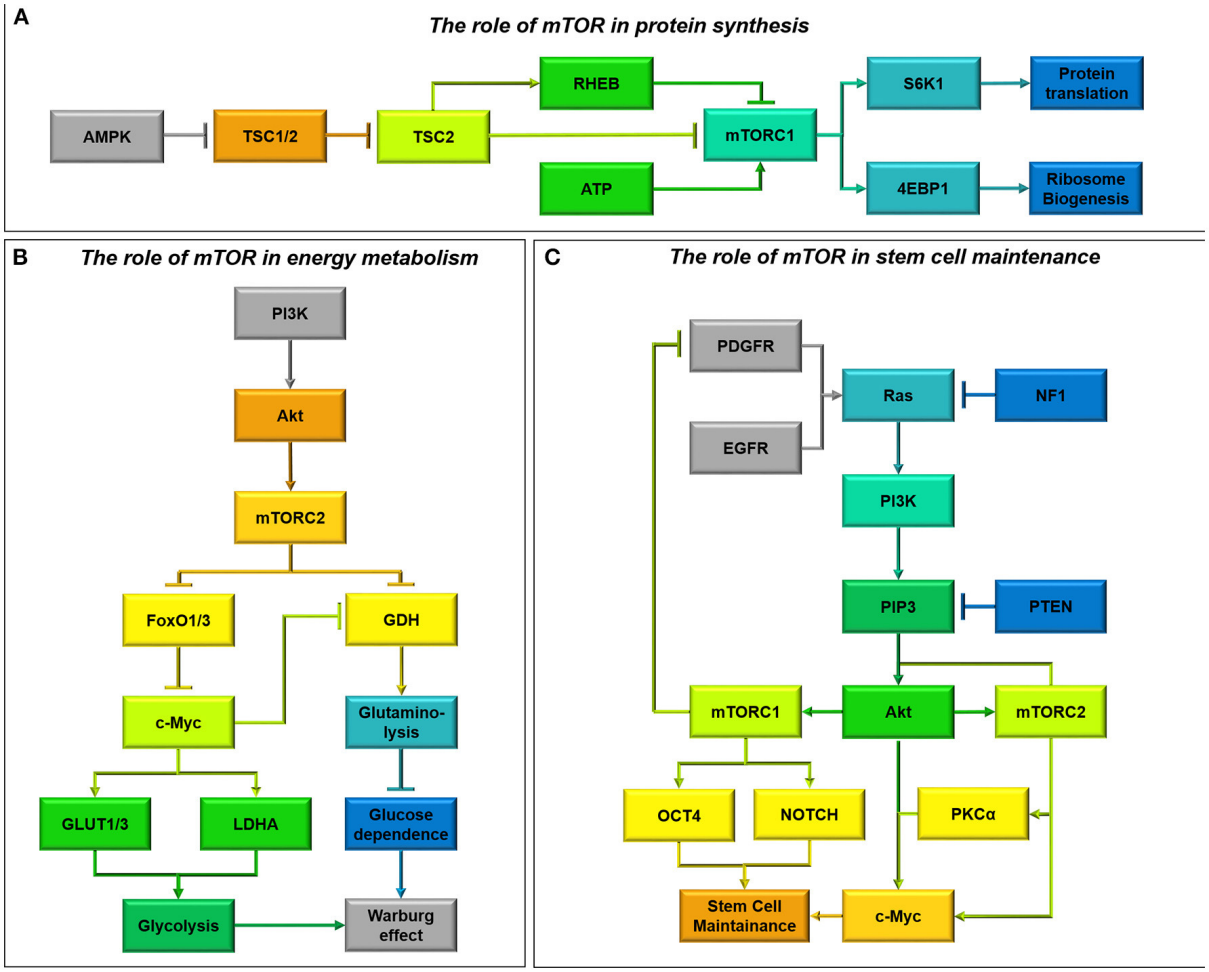
**(A)** The role of mTOR in protein synthesis. AMPK is a well-known regulator of mTOR activity (Vucicevic et al., 2011). Upon activation AMPK destabilizes the Tumor Suppressor 1/2 (TSC1/2) complex, allowing TSC2 to freely bind Ras-homolog enriched in brain (RHEB) and inhibit mTORC1 signaling (Laplante and Sabatini, 2012). This means that energy consuming processes such as protein translation and ribsome biogenesis are inhibited during periods of energy stress. As well as acting through AMPK, ATP has recently also been shown to be able to directly activate mTORC1 signaling (Dennis et al., 2001). **(B)** The role of mTOR in energy metabolism. mTORC2 metabolic reprogramming downstream of PI3K/Akt is a critical regulator of the Warburg effect and glucose dependence in glioma cells (Masui et al., 2015). By relieving FoxO1/3 constraint on c-Myc signaling, mTORC2 increases Glucose Transproter 1 and 3 expression (GLUT1/3) as well as lactate dehydrogenase activity, encouraging aerobic glycolysis (Masui et al., 2013). Simultaneously, through c-Myc activity mTORC2 also inhibits glutamate dehydrogenase (GDH) activity required for glutaminolysis, regulating a metabolic switch from oxidative phosphorylation fuelled by the Kreb's cycle to aerobic glycolysis (Yang et al., 2009). mTORC2 signaling therefore causes glioma cells to become “addicted” to aerobic glycolysis, making them particularly vulnerable to glucose depletion. **(C)** The role of mTOR in stem cell maintenance. Oncogenic activity which converges on PI3K can activate mTOR signaling (Sarbassov et al., 2005). mTORC1 and mTORC2 activity results in the activation of several transcription factors, including OCT4 and NOTCH and c-Myc respectively, ensuring stem cell maintainence (Masui et al., 2013). c-Myc can also be activated directly by Akt signaling and PKCα signaling downstream of mTORC2 to induce stem cell transformation (Fan et al., 2009). Additionally, whilst mTORC2 activity forms part of a feed-forward system, phosphorylating Akt, mTORC1 activity downregulates PI3K activity by downregulating PDGFR (Akhavan et al., 2010; Sarbassov et al., 2005).

AMPK is a well-known regulator of mTOR. AMPK destabilizes the TSC1/2 complex, allowing TSC2 to bind RHEB stimulating its GTPase activity and inhibiting its ability to activate mTORC1 (Vucicevic et al., 2011). Interaction with AMPK allows mTORC1 to sense energy levels and p53 status, however it has recently been hypothesized that mTORC1 activity may also be directly influenced by ATP (Dennis et al., 2001; Levine and Puzio-Kuter, 2010). Low amino acid levels can attenuate mTORC1 activity by inhibiting Rag GTPases bound to lysosomes, thereby inhibiting mTORC1 recruitment and activation by RHEB (Laplante and Sabatini, 2012). In this manner mTOR regulates anabolic processes by inducing protein synthesis in response to substrate levels (Figure 8A).

### mTOR as a regulator of the warburg effect in glioma

Although mTORC1 controls protein synthesis and cell cycle entry, the metabolic effects of mTOR activation are regulated by mTORC2. In serum-supplemented U-87 cells, mTORC2 acts upstream of c-Myc, another critical metabolic regulator which induces the Warburg effect (Masui et al., 2013; Figure 8B). mTORC2 controls c-Myc by inhibiting FoxO1 and FOX03, through phosphorylation of PKCα and inhibition of class IIa histone deacetylases which phosphorylate and acetylate FoxO (Masui et al., 2013, 2015). This allows transcription of glycolytic pathway genes, upregulation of glucose transporters (GLUT1/3) and increased lactate production (LDHA) in U-87 cells (Masui et al., 2013; Clark et al., 2016). Akt-expressing cells are also able to induce glycolysis through direct phosphorylation of FoxO1/3, relieving the blockade on c-Myc signaling and facilitating glycolysis (Yang et al., 2009; Masui et al., 2015; Figure 8B).

Akt and mTORC2 signaling confer glucose addiction within glioma cells both *in vitro* and *in vivo* (Yang et al., 2009; Tanaka et al., 2015), and without mTORC2 activity U-87 cells cannot sustain their proliferation in glucose (Masui et al., 2013). Therefore, impairments in glucose availability can be devastating for glioma survival. Glioma cells upregulate glutamine metabolism as a compensatory mechanism to sustain flux through the Kreb's cycle, due to the ability of glucose withdrawal to stimulate glutamate dehydrogenase activity (Yang et al., 2009). This upregulation also aids in protecting SF-188 and U-87 cells against Akt and mTOR inhibition, by relieving the suppression of glutamate dehydrogenase and glutaminase by Akt and inducing α-KG-dependent anaplerosis (Yang et al., 2009; Tanaka et al., 2015). Therefore, dual inhibition of mTOR and glutaminase has proved to be effective in immunodeficient subcutaneous xenografts of U-87 cells (Tanaka et al., 2015). AMPK activation has also been shown to protect against glucose withdrawal in Akt-expressing cells, by reducing the cellular ability to induce beta-oxidation (Buzzai et al., 2005).

### Role of mTOR in resistance of glioma to therapy

Persistently activated PI3K/Akt/mTOR axis signaling is associated with the development of cancer (Cancer Genome Atlas Research Network, 2008, Figure 8C). For maximal Akt signaling and proliferation in cancer cells, Akt is phosphorylated by both PI3K induced kinases at Thr308 and by mTORC2 at Ser473 (Akhavan et al., 2010). Interestingly, mTORC2 activation of PKCα can propagate mTORC1 signaling through Akt activation (Sarbassov et al., 2005; Fan et al., 2009). Due to this mTORC2 is an essential component for tumor growth in response to enhanced EGFR signal flux through PI3K in glioma (Read et al., 2009), conferring resistance to EGFR inhibitors (Stommel et al., 2007).

mTOR has been shown to mediate at least some of the effects of concurrent loss of NF1 and PTEN (Verhaak et al., 2010, Figure 8C). Loss of NF1 and p53, is sufficient to achieve gliomagenesis in an mTOR-dependent process, where inhibition of mTOR reduces NF1-mediated progression in mouse models of optic glioma (Galvao et al., 2014; Kaul et al., 2015). Meanwhile, cells with wild-type PTEN have reduced mTORC2 activation through Rictor Thr1135 phosphorylation; mutation or depletion of PTEN prevents this inactivation, allowing mTORC2 activation and reduced cell cycle arrest (Bhattacharya et al., 2016, Figure 8A).

Sustained mTORC2 signaling, due to augmented p53, constitutive EGFRvIII signaling, and NF1/PTEN co-deletion can confer resistance to a variety of drugs used to treat glioma. PI3K and Akt inhibitors and Rapamycin are commonly used to block the PI3K/Akt/mTOR axis, however rapamycin-insensitive and sustained mTORC2 signaling imparts survival in U-87 cells (Masui et al., 2013). Although PI3K and Akt inhibition would be expected to deplete c-Myc levels, mTORC2 increases FoxO acetylation as a compensatory mechanism to modulate c-Myc and promote GBM survival (Masui et al., 2013). Due to this signaling complexity mTORC2 has been highlighted as a major regulator of GBM growth and drug resistance (Masui et al., 2015). As mTORC2 knockdown suppresses the induction of glycolysis in response to PI3K and Akt inhibitors, it has been proposed that their combination with dual mTORC1/2 inhibitors will promote glioma cell death and tumor regression (Masui et al., 2013).

## P53 mutations affect not only cell cycle checkpoints but also cellular bio-energetics

Due to the interaction between energy metabolism and proliferation, it is unsurprising that proteins known to control the cell cycle also have a profound effect on metabolism (and vice-versa). Within glioma p53 has a varied role, retaining its wild-type conformation in most primary glioblastomas and acquiring gain-of-function mutations in the pro-neural glioblastoma subtype and during lower-grade glioma progression (Guo et al., 2012; Wanka et al., 2012a). p53 is widely known as a tumor suppressor, acting upstream of many oncogenic nodes. p53 also has the effect of restricting aerobic glycolysis and promoting oxidative phosphorylation, while loss of p53 function contribute to the Warburg effect (Levine and Puzio-Kuter, 2010).

p53 promotes responses to extrinsic and intrinsic stimuli dependent on the type, severity and persistence of stress (Vousden and Prives, 2009). Whilst wild-type p53 expression is associated with decreased proliferation due to inhibition of cyclin-dependent kinases, overexpression of regulatory mechanisms such as MDM2 control p53-dependent growth in glioma (Reifenberger et al., 1993; Suh et al., 2012). MicroRNA-25 and -32 are repressed by p53-dependent activities as part of a negative feedback loop in U-87 cells, however their suppression also alleviates the block on MDM2 activity which ubiquitinates and degrades p53 (Xirodimas et al., 2001; Suh et al., 2012). Expression of miR-25 and -32 in U-87 cells with functional p53 inhibits their growth *in vivo*, signifying the importance of this autoregulatory feedback loop in GBM proliferation (Suh et al., 2012).

### Induction of oxidative metabolism by p53

By inhibiting key oncogenic pathways that promote aerobic glycolysis as discussed in previous sections, p53 is able to constrict glycolytic respiration both indirectly and through its direct interaction with glycolytic pathway components (Deberardinis et al., 2008, Figure 9A). p53 represses the transcription of GLUT1/4 transporters and ChREBP leading to decreased glycolysis in colorectal cancer cell lines (Dang, 1999; Tong et al., 2009). Whereas, the p53 product TIGAR, often overexpressed in glioblastomas, protects against glycolytic adaptation by reducing the expression and activity of Phosphofructokinase-1 and fructose biphosphatase in multiple cancer types, including GBM (Bensaad et al., 2006; Wanka et al., 2012b).

**Figure 9:**
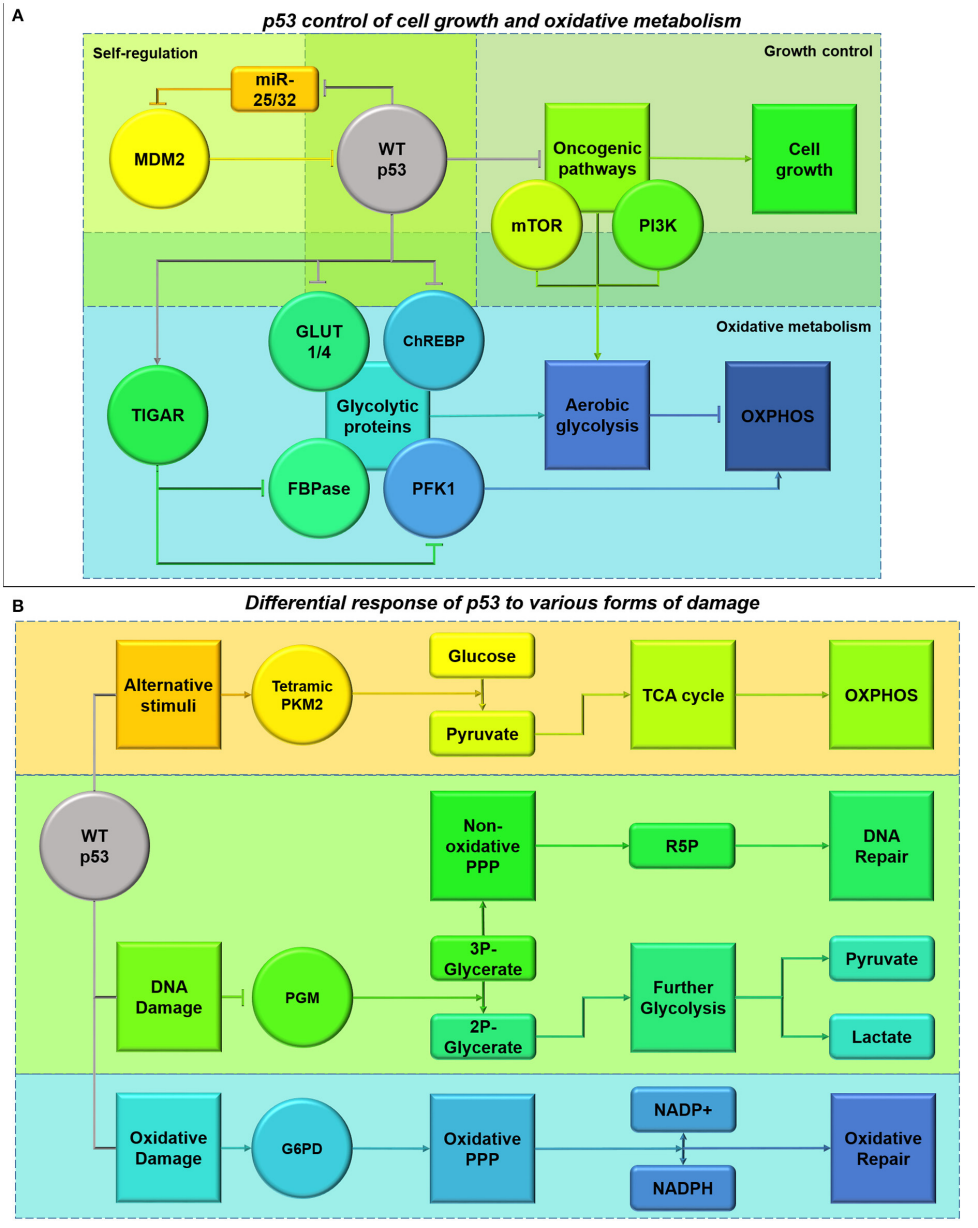
**(A)** Functional p53 control of cell growth and oxidative metabolism. p53 plays a role in its own regulation by diminishing the inhibition of MDM2 which mediates p53 ubiquitination, through a feedback loop involving miR-25 and -32 (Suh et al., 2012). p53 also has a role in inhibiting the activity of oncogenic pathways, including mTOR and PI3K/Akt, halting cell cycle progression whilst DNA is repaired (Budanov and Karin, 2008). This cell cycle blockade can be alleviated by MDM2 (Reifenberger et al., 1993). This also alleviates the glycolytic switch inferred by the pathways. Additionally, p53 also has its own roles in diminishing glycolysis both directly and indirectly through TIGAR transcription (Bensaad et al., 2006). **(B)** Differential response of p53 to different forms of damage. Activation of p53 due to DNA damage (shown in green), results in the suppression of phosphoglycerate mutase (PGM) causing inhibition of the glycerate reduction. This process allows activation of the non-oxidative arm of the PPP and production of R5P for DNA repair, instead of channeling glycolytic intermediates through the glycolytic pathway (Kondoh et al., 2005; Deberardinis et al., 2008; Levine and Puzio-Kuter, 2010). Additionally, activation of p53 by oxidative damage (shown in blue) inhibits the degradation of G6P-dehydrogenase (G6PD), stimulating the oxidative PPP phase and NADPH production to counter-act ROS and oxidation (Jiang et al., 2011). These pathways provide protection from extensive DNA and oxidative damage in cancer cells expressing functional p53. However, under conditions where p53 has been activated in response to other stimuli such as energy deprivation (shown in orange), p53 can inhibit these pathways to free-up glycolytic intermediates for pyruvate formation and Kreb's cycle entry (Boros et al., 1997; Christofk et al., 2008).

Functional p53 increases expression of glutaminase 2 (GLS2) under stress in HTB-15 human glioblastoma cells (Hu et al., 2010). This serves to increase oxidative metabolism and ATP generation, by catalyzing the conversion of glutamine to glutamate and increasing α-KG levels (Hu et al., 2010). This metabolic effect is also seen in freshly isolated human GBM stem cells (Michelakis et al., 2010). Additionally, increased glutamate availability as a precursor for glutathione (GSH) helps to protect against oxidative stress (Hu et al., 2010; Michelakis et al., 2010). Currently the impact of wild-type and gain-of-function p53 mutations on rates of oxidative metabolism in GBM is relatively understudied. However, in other cancer cell types p53-mediated transcription of hexokinase 2 catalyzing the first step of glycolysis and the production of tetrameric PKM2 converting glucose to pyruvate, result in greater oxidative metabolism (Mathupala et al., 1997; Christofk et al., 2008, Figure 9B). p53 also supports the expression of PTEN, influencing the switch from aerobic glycolysis to oxidative metabolism by inhibiting the PI3K/Akt/mTOR pathway (Stambolic et al., 2001). Therefore, it is expected that p53 mutations reduce oxidative metabolism, given our current understanding of p53 function; gain-of-function mutations may drive an oxidative metabolic phenotype.

AMPK and p53-dependent signaling act in concert to halt cell cycle progression whilst restoring energy homeostasis (Jones et al., 2005). Metformin, which acts to stimulate AMPK activity, causes synthetic lethality in colon carcinoma cells with p53 mutations highlighting the close interaction between these proteins (Buzzai et al., 2007). p53 plays a role in increasing mitochondrial complex activity due to AMPK activation by enhancing the transcription of COX assembly protein 2 (SCO2) (Matoba et al., 2006; Deberardinis et al., 2008). SCO2 in combination with SCO1, forms COX acting as the last enzyme within the electron transport chain, increasing oxygen consumption and ATP generation (Matoba et al., 2006). To increase flux into the Kreb's cycle, p53 increases GLS2 expression, in opposition to c-Myc which decreases Kreb's cycle activity by enhancing GLS1 expression (Hu et al., 2010; Suzuki et al., 2010). Within glioblastoma cells p53 induction results in increased GLS2 under oxidative stress but not oxygen or nutrient starvation (Hu et al., 2010). However, basal p53 activity also results in 2.5-3-fold increased GLS2 transcription, maintaining glutamate and α-ketoglutarate levels (Hu et al., 2010). In contrast, during nutrient deprivation malate dehydrogenase 1 interacts with p53 to modulate its transcriptional targets within glioma cells to maintain energy homeostasis (Lee et al., 2009). Therefore, cancer cells without functional p53 are particularly sensitive to nutrient deprivation, diminishing the scope for engaging catabolic pathways such as autophagy and beta-oxidation in glioma (Buzzai et al., 2007; Munoz-Pinedo et al., 2012).

By interacting with pathways such as PI3K and mTOR, p53 is also able to limit HIF complex stabilization, resulting vascularization and migration (Budanov and Karin, 2008). Additionally, p53 is also able to maintain ROS homeostasis limiting the effect of these molecules on cell signaling dynamics (Budanov et al., 2004). p53 activation mediates ROS levels by inducing Sestrins1-4 and regulating p21, increasing ROS detoxification and Nrf2 antioxidant capabilities respectively (Mathupala et al., 1997; Budanov et al., 2004, Figure 9B). The induction of Sestrins 1 and 2 also aids in stimulating AMPK activity, as part of a constitutive feedback mechanism for p53 induction under oxidative stress (Budanov and Karin, 2008). Through increasing GLS2 expression, p53 is also able to increase *de novo* GSH production and maintain GSH/GSSG (GSH disulphide) ratios in response to oxidative stress in glioblastoma cells by increasing GSH pre-cursors (Hu et al., 2010; Suzuki et al., 2010). Due to this increased resistance to oxidative stress, human glioma cells with functional p53 also show greater resistance to gamma-radiation, by reducing the toxic production of ceramide and resulting apoptotic induction (Hara et al., 2004, Figure 9B).

Suppression of p53 in glioblastoma cells enhances the effects of hypoxia and susceptibility to hypoxia-induced apoptosis (Wanka et al., 2012a). A recent study highlighted increased protection from moderate hypoxia (1% O_2_) correlates with p53 induction of SCO2, which is necessary for complete respiratory chain function in human glioma (Wanka et al., 2012a). This helps to maintain oxidative phosphorylation and resistance to hypoxia after p53 ablation, however this protective effect is not seen under profound hypoxia (0.1% O_2_) (Wanka et al., 2012a). In this manner p53 may provide a significant survival advantage for glioma cells, potentially accounting for wild-type p53 retention in primary gliomas. SCO2 deficiency reduces the number of islets of viable cells in necrotic areas of colon carcinoma *in vivo*, however this viability is retained in p53 expressing tumors conferring a unique survival advantage (Wanka et al., 2012a).

### Role of p53 in response of glioma cells to therapy

During chemotherapy p53 also provides some protective effects by inducing the transcription of genes involved in melavonate pathway, alleviating the brake on cholesterol biosynthesis (Mo and Elson, 2004). p53 induces the expression of several proteins involved in melavonate production such as HMGCR and LDLR (low-density lipoprotein receptor), which are upregulated at basal levels in GBM cells compared to normal astrocytes (Laezza et al., 2015). This may represent another pathway by which functional p53 may be able to overcome proliferative defects, as cholesterol is required for DNA synthesis and proliferation. However, loss of p53-dependent control of LDLR and another melavonate pathway element, RabGGTA, often accompanies transformation (Laezza et al., 2015). Whilst this represents a problem for maintaining proliferation by reducing total cellular cholesterol pools, loss of p53 control is overcome by endogenous cholesterol synthesis (Laezza et al., 2015).

Wild-type p53 in U-87 cells reduces recovery in response to ionizing radiation, whereas mutant p53 in T98 cells demonstrate robust proliferation and reduced induction of senescence (Quick and Gewirtz, 2006). The presence of functional p53 in U-87 cells enhances temozolomide response by inducing apoptosis upon prolonged G_2_-M arrest (Hirose et al., 2001a,b). Whilst glioma cells expressing functional p53 show both G1 and G2/M blocks to a limited degree, p53 null cells demonstrate a prominent G2/M block which plays a role in radio-resistance (Tsuboi et al., 2007). This may also explain why p53 is depleted in secondary lesions; natural selection may be at play, favoring the clonal expansion of p53-mutated cells. Induction of wild-type but not mutant p53 reduces angiogenic activity of the malignant glioma cell line LN-Z308 (Van Meir et al., 1994). Surprisingly, sustained impairments of functional mitochondrial metabolism in neural progenitor cells can also lead to p53 genetic inactivation through increased ROS production and resulting damage to DNA (Bartesaghi et al., 2015). Although this is a relatively new concept, it may help to explain p53 loss during glioma progression, whereby mitochondrial dysfunction potentially damages the cell, conferring protection against radiotherapy.

## Peroxisome proliferator-activator receptors are central regulators of gene transcription and oxidative metabolism

**Figure 10:**
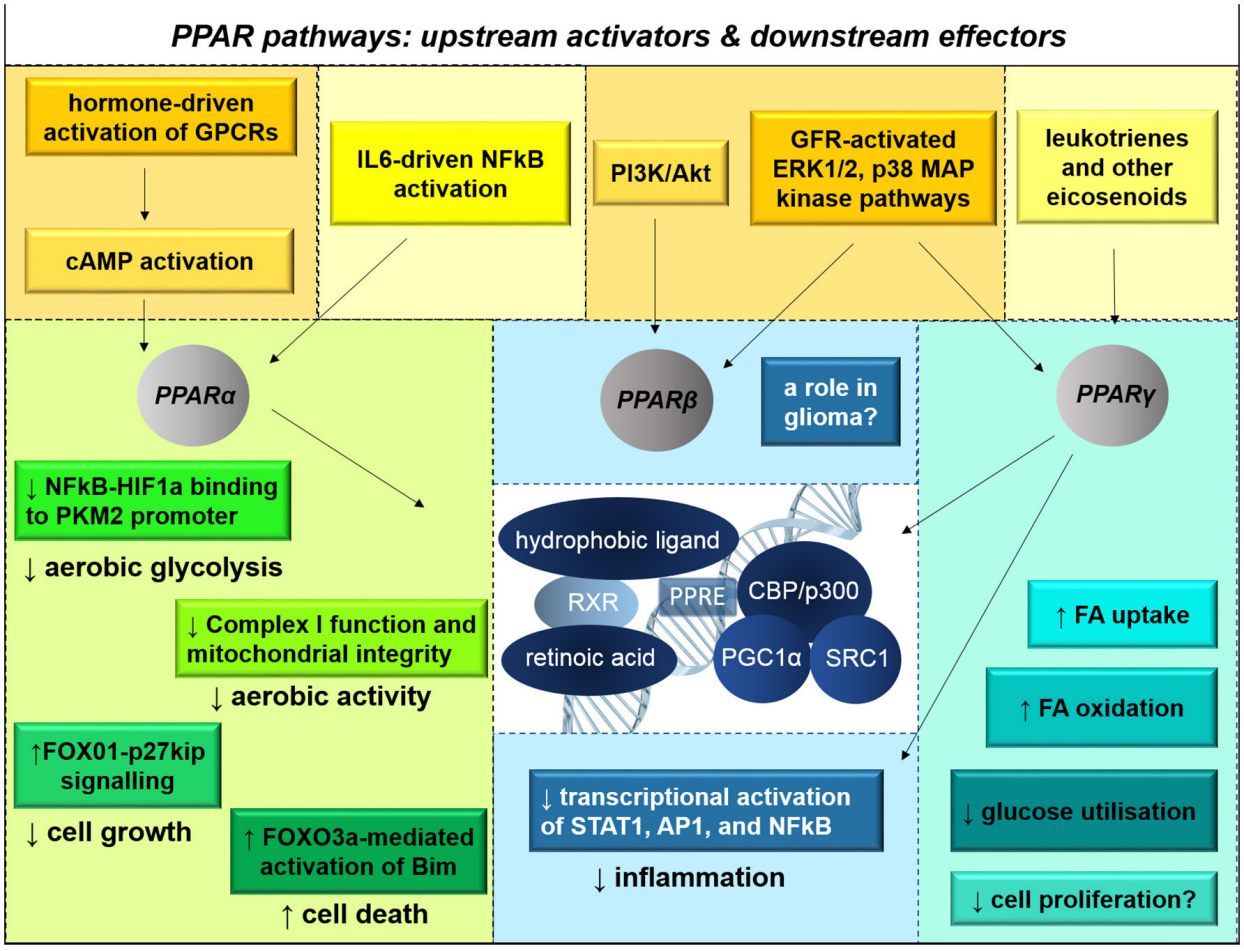
**PPAR signaling pathways influence metabolic capacity, growth, and survival**. There are three subtypes of peroxisome proliferator-activated receptor proteins (PPARs). PPARs are nuclear hormone receptors that are activated primarily by eicosanoids, unsaturated fatty acids, NSAIDS, and retinoic acid. Their activation status is also influenced by cAMP second messenger cascades and MAP kinase signaling activated by G-protein-coupled receptors (GPCRs) and growth factor receptor (GFR)-activated signaling. Upon heterodimerization with retinoic acid receptors (RXRs) and association with hydrophobic ligands, PPARs translocate to the nucleus. There, the complexes activate transcription of metabolic genes. PPARα agonism disrupts HIF1α-mediated transcriptional activation of PKM2, thereby reducing glycolysis; PPARα has also been implicated in tumor suppression and apoptotic initiation through various signaling pathways. PPARγ enhances mitochondrial biogenesis, fatty acid oxidation, and insulin-mediated glucose transport into cells; PPARγ is also anti-inflammatory, preventing the activation of STAT1, AP1, and NFκB. In many contexts, non-ligand-activation of PPAR induces transcriptional repression. The effects of PPARβ signaling in glioma are not known.

Peroxisome proliferator-activated receptor proteins (PPARs) are nuclear receptors; the three subtypes (PPARα, PPARβ, and PPARγ) exert different effects on cellular behavior, although broadly do so by impinging on metabolic gene transcription (Figure 10). PPARs are activated by various signaling pathways, including cAMP second messenger cascades downstream of G-protein-coupled receptors and growth factor receptor-activated MAP kinase signaling pathways, thus well-placed to link extracellular factor-induced signaling pathways with cellular metabolic activity. PPARs bind to hydrophobic ligands including eicosanoids, unsaturated fatty acids and retinoic acid, and interact with retinoic acid receptors and cofactors including PGC1α to form transcription factor complexes (Tontonoz and Spiegelman, 2008). Upon activation, PPARs translocate to the nucleus and bind to PPAR response elements. Although PPARα and PPARγ bind to the same cofactors, they appear to have differential effects on gene transcription in glioma cells; the role of PPARβ in glioma has not been characterized.

### PPAR-alpha

PPARα expression is lower in high-grade gliomas compared with normal brain tissue; signifying worse prognosis in GBM patients. PPARα overexpression inhibits growth, invasion, and aerobic glycolysis in glioma cells (Shi et al., 2016). Fenofibrate, a PPARα agonist which drives transcriptional activity, has been shown to reduce cancer cell growth in U-87-MG glioma cells and in other cancers (Saidi et al., 2006; Panigrahy et al., 2008; Han D. F. et al., 2015). Fenofibrate lowers lipid content in the bloodstream; it also directly inhibits glycolysis in glioma cells by disrupting binding of the NF-κB-HIF1a complex to the PKM2 promoter (Han D. et al., 2015). This drug also causes structural damage to mitochondria and inhibition of Complex I, leading to activation of the AMPK-mTOR pathway, promoting autophagy (Wilk et al., 2015; Han D. et al., 2015). The broad-ranging metabolic effects of fenofibrate on glioma cells are associated with lowered cell growth, due to FOX01/p27-induced G0/G1 arrest, and increased apoptotic cell death caused by FOXO3a-activated transcriptional activation of the apoptotic initiator protein Bim (Wilk et al., 2012; Han D. F. et al., 2015).

### PPAR-gamma

The nuclear receptor PPARγ is activated by hydrophobic molecules including fatty acids (Tontonoz and Spiegelman, 2008). Once activated by a ligand, the PPARγ complex binds to response element sequences throughout the genome and modulates transcription of its target genes, many of which enhance mitochondrial function and beta-oxidation (Itoh et al., 2008). Over-representation of PPARγ polymorphism H449H is observed in sporadic cases of glioblastoma compared with the normal population (Zhou et al., 2000); however the functional significance of this is not known. PPARγ agonists cause upregulation of the glutamate transporter, reducing extracellular glutamate levels and excitotoxicity in the vicinity of in U-87 and U-251 cells (Ching et al., 2015). In addition, agonists of PPARγ reduce tumor growth in xenografted LN-229 cells (Grommes et al., 2013), however it should be noted that antagonism of PPARγ conversely reduces tumor growth in Sonic hedgehog-driven mouse models of medulloblastoma (Bhatia et al., 2012). PPARγ agonists have therefore been proposed as novel anti-neoplastic agents for the treatment of glioma, although currently these drugs have not been evaluated in serum-free glioma cell cultures or direct-to-xenograft animal models (Ellis and Kurian, 2014). A Phase II clinical trial evaluating treatment with pioglitazone, a relatively specific PPARγ agonist, in combination with the chemotherapeutic temozolomide and the COX2 inhibitor rofecoxib, did not demonstrate improved prognosis, and individual patient responses did not correspond to protein expression levels of COX2 or PPARγ (Hau et al., 2007). More studies are needed to evaluate the biological and functional roles of PPARs in glioma.

### Sirtuins

Another group of regulatory proteins which acts independently and in partnership with PPAR nuclear receptors are the sirtuins, which are Class III histone deacetylases. Sirtuins provide a direct connection between catabolic activity and histone deacetylation in cancer cells. High NAD+ levels activate Sirtuins, which in turn repress expression of key tumor suppressor genes while increasing expression of telomerase (Zhang et al., 2014). These proteins also modulate a number of signaling pathways to influence cellular metabolism and oncogenic potential. In particular, Sirtuin-1 has been shown to inhibit HIF activation, activate mitochondrial biogenesis through PPARγ-cofactor 1α (PGC1α), drive lipid metabolism through sterol regulatory element-binding protein factors, mediate inflammatory signaling through NF-kB, and inhibit p53-mediated apoptosis through inhibitory deacetylation in other cell contexts (Lavu et al., 2008; Kitada et al., 2013; Gonzalez Herrera et al., 2015). This family of proteins have not been well-studied in the context of glioma, although tantalizing initial evidence suggests that Sirtuin-1 is actually required for gliomagenesis (Lee et al., 2015) and inhibition of Sirtuin-1 through miR-22 (which also targets EGFR and matrix metalloprotease 9) slows the growth and invasiveness of U-87 and U-251 glioma cells (Chen et al., 2016). Further studies are needed to identify the roles of Sirtuin-1 and other sirtuins in the epigenetic modulation of glioma cells, and how these effects might be mediated.

## Reactive oxygen species and redox homeostasis

### Production of reactive oxygen species (ROS) in glioma cells

The electron transport chain couples the transfer of charge across the inner mitochondrial membrane with the production of ATP. The leakage of ROS and protons from Complexes I-IV of the electron transport chain represents a self-regulating system to reduce oxidative stress, whereby ROS themselves induce proton leak and decrease ROS generation (Brookes, 2005). Redox homeostasis involves the Nrf2-dependent antioxidant system which is important for inhibiting differentiation and promoting drug resistance in GSCs (Akhavan et al., 2010; Cardaci and Ciriolo, 2012). GSH acts as an electron acceptor to reduce ROS during its conversion to GSSG (Levine and Puzio-Kuter, 2010). GSH is then reformed from GSSG by GSH reductase which reduces NADPH to form NADP+ (reformed during the PPP and Kreb's cycle) (Levine and Puzio-Kuter, 2010; Wanka et al., 2012b, Figure 11A). Oxidative stress is also avoided via ROS scavengers: glutathione peroxidase (GPx), superoxide dismutases (SODs), catalase, thioredoxin, and peroxiredoxin (Jin et al., 2015).

**Figure 11:**
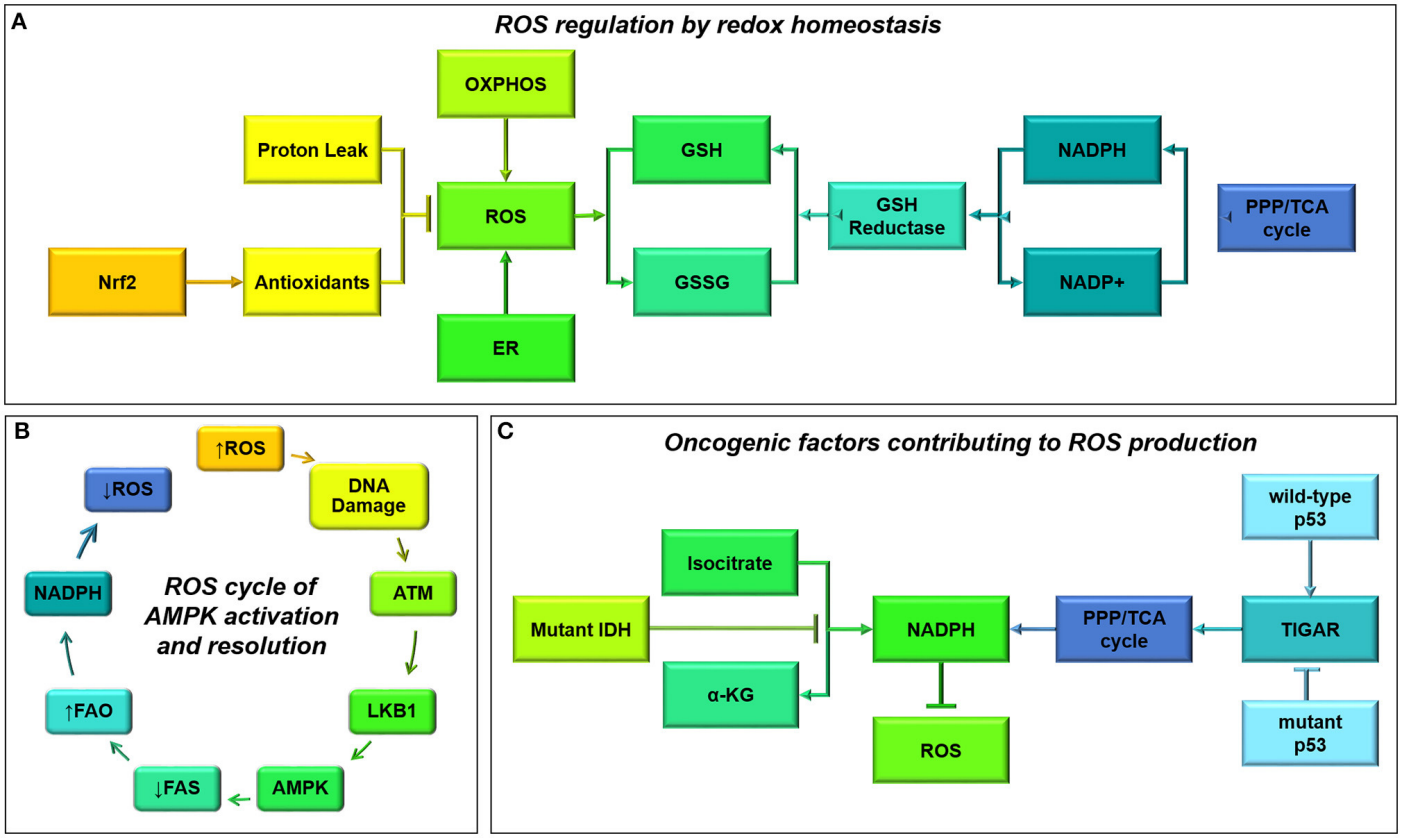
**(A)** Reactive oxygen species (ROS) maintenance by redox homeostasis. Oxidative phosphorylation and endoplasmic reticulum (ER) activity both result in the formation of ROS (Salazar-Ramiro et al., 2016). These ROS can be neutralized by antioxidants, under the transcriptional control of Nrf2, the master regulator of ROS homeostasis (Cardaci and Ciriolo, 2012). Additionally, proton leak in the mitochondria also helps to neutralize ROS through a self-regulating system (Brookes, 2005). Another system to regulate ROS levels, acts through the glutathione (GSH) system which neutralizes ROS upon conversion into glutathione disulphide (GSSG) (Levine and Puzio-Kuter, 2010). Within cells, GSH levels are maintained by production of NADPH from the PPP and Kreb's cycle which maintain GSH reductase activity (Levine and Puzio-Kuter, 2010). **(B)** ROS cycle of AMPK activation and resolution. Increased ROS levels result in oxidative DNA damage and the activation of the DNA damage response pathway by ataxia-telangiectasia mutated (ATM) recognition (Alexander and Walker, 2011). This stimulates LKB1 activity, and increased phosphorylation and stimulation of AMPK. As a result AMPK inhibits NADPH consuming pathways such as FAS and activates catabolic NADPH-producing pathways such as FAO, which acts through the GSH/GSSG antioxidant system to neutralize ROS and restrain further oxidative damage (Jeon et al., 2012). **(C)** Oncogenic factors contributing to ROS production. Two common mutations in secondary gliomas include the mutation of isocitrate dehydrogenase (IDH) and p53 pathways (Cuperlovic-Culf et al., 2012). Through their downstream activities, i.e., IDH reducing isocitrate into α-KG and p53-mediated transcription of TIGAR, these proteins aid in maintaining NADPH levels and subsequent ROS neutralization (Wanka et al., 2012b; Klink et al., 2016). However, upon mutation these pathways are inhibited resulting in a loss of NADPH, increased ROS and a higher rate of oxidative damage within glioma cells.

Although cancer cells rely heavily on glycolysis and glutaminolysis to reduce ROS production, oxidative phosphorylation does contribute to the ATP pool (Zhang et al., 2015; Ozcan and Cakir, 2016). In the rapidly proliferating SF-188 pediatric glioblastoma cell line there is a shift away from glycolysis toward oxidative glutamine metabolism represented by increased oxygen consumption and increased ROS (Pike Winer and Wu, 2014); likewise adult glioblastoma cells are also highly oxidative (Lin et al., 2017). Additionally endoplasmic reticulum activity involving protein and lipid formation, contributes 25% of cellular ROS production in glioma cells (Salazar-Ramiro et al., 2016). Increased ROS levels contribute to a variety of tumorigenic processes inducing proliferation, genetic instability and evasion from senescence (Levine and Puzio-Kuter, 2010). Cancer cells show an increased tolerance for oxidative stress whereby moderate ROS levels promote proliferation and differentiation, whilst excessive ROS exposure causes oxidative damage and induces apoptosis (Jin et al., 2015; Rinaldi et al., 2016).

### Role of AMPK in redox homeostasis

The LKB1/AMPK axis represents another system by which cells control redox homeostasis (Figure 11B). Upon ROS-mediated DNA damage, LKB1 associates with ATM kinase and AMPK, resulting in phosphorylation of ACC (Alexander and Walker, 2011; Jeon et al., 2012). By regulating the switch from anabolism to catabolism, AMPK stimulates NADPH production through beta-oxidation limiting oxidative stress (Jeon et al., 2012). LKB1 is also able to restrict oxidative damage through its AMPK-independent interaction with Cdc42 and decreased phosphorylation of p38-MAPK reducing anabolic ROS production (Xu et al., 2015).

Mutations in the tumor suppressor LKB1 limit AMPK activity and the production of NADPH through catabolic processes; unrestrained ROS can promote tumor growth by boosting oncogenic signal transduction, genetic instability and glioma growth *in vivo* (Jeon et al., 2012). ROS are also able to inhibit LKB1-mediated activation of AMPK, alleviating mTOR inhibition and promoting entry into the cell-cycle and glioma proliferation *in vitro* (Jiang et al., 2014). Downstream of AMPK, PGC-1α controls transcription of antioxidative proteins alongside mitochondrial biogenesis, highlighting the tight control of ROS in response to increased mitochondrial respiration (Hartel et al., 2016). This system aids glioma cells in evading apoptosis due to prolonged ROS exposure.

### Role of ROS in glioma development

IDH plays a role in producing antioxidants, as well as promoting oxidative respiration and oxygen-sensitive signaling transduction (Cuperlovic-Culf et al., 2012, Figure 11C). Under normal circumstances IDH activity reduces NADP+ whilst catalyzing the oxidative decarboxylation of isocitrate to α-ketoglutarate, however IDH mutants consume NADPH during the synthesis of the oncometabolite 2-hydroxyglutarate, thereby decreasing antioxidant defense (Cuperlovic-Culf et al., 2012; Lewis et al., 2014; Klink et al., 2016). The central role of a functioning Kreb's cycle in maintaining redox balance is highlighted by conversion of glutamate into α-KG by glutamate dehydrogenase and the maintenance of NADPH and GSH/GSSG ratios (Jin et al., 2015). TIGAR is also involved in protecting glioma cells from metabolic and oxidative stress (Figure 11C). Through enhancing the energy yield from glucose and promoting oxidative respiration, TIGAR inhibits ROS production through enhancing NADPH and GSH:GSSG ratio as a result of enhanced PPP flux (Wanka et al., 2012b).

EGFR activation or constitutive EGFR signaling induce ROS production in glioma cell lines (Salazar-Ramiro et al., 2016). Additionally, glioma cells exhibiting loss of the tumor suppressor p53 also display higher levels of ROS and oxidative stress due to the loss of SOD2, GPX1, and ALDH4A1, which form part of the antioxidant system (Macedo et al., 2012). ROS can also directly phosphorylate signaling proteins and increase flux through PI3K/Akt and MAPK/ERK to potentiate oncogenic signaling (Boonstra and Post, 2004; Rinaldi et al., 2016, Figure 12). Interestingly, increased ROS in GSCs is associated with reduced self-renewal, increased cycling, and reduced viability through activation of p38 under oxidative stress (Yuan et al., 2015). However, under non-stressed conditions ROS-activated JNK and p38 cooperate with ERK to support proliferation and increase tumorigenic activity in glioma (Benhar et al., 2002; Yuan et al., 2015).

**Figure 12:**
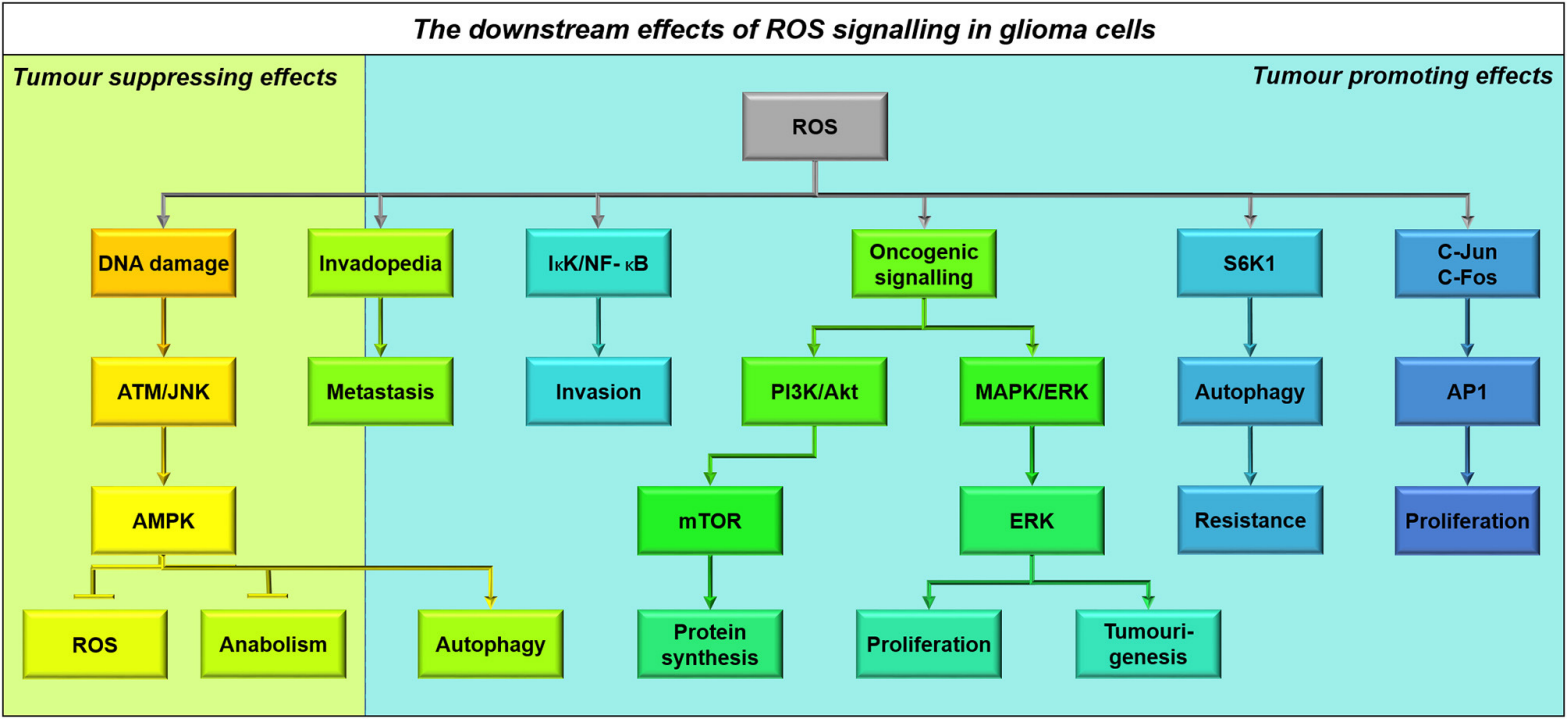
**The downstream effects of ROS signaling in glioma cells**. Activation of AMPK as a result of oxidative damage induced by ROS, has both tumor suppressive and promotive effects within glioma cells, through AMPK signaling (Alexander and Walker, 2011). Additionally ROS aids in oncogenic signaling resulting in the activation of pathways involved in metastasis, invasion, proliferation, and resistance (Boonstra and Post, 2004). ROS mediates the formation of invadopedia involved in metastasis and inflammatory signaling through NF-kB to promote matrix degradation and invasion (Zhang et al., 2015). Additionally PI3K/Akt and MAPK/ERK pathways are also augmented resulting in increased proliferation, as well as increasing c-Jun and c-Fos formation of the activator protein-1 (AP-1) transcription factor involved in proliferation (Benhar et al., 2002; Waris and Ahsan, 2006). Finally, ROS are also involved in S6K1 activation which acts to induce autophagy providing chemotherapeutic resistance (Sarbassov and Sabatini, 2005).

Redox status also regulates responses to nutrient deprivation. Oxidizing agents which reduce the NADPH/NADP+ ratio, and increase ROS, can increase mTORC1 signaling and confer resistance to nutrient deprivation and inhibition of the PI3K/Akt pathway (Sarbassov and Sabatini, 2005). Reducing agents act in the opposite manner to mimic cellular responses to nutrient deprivation, slowing proliferation. Additionally, ROS can also directly phosphorylate c-Jun and c-Fos, resulting in activation of activator protein-1 transcription factor, glioma growth and proliferation (Waris and Ahsan, 2006). In glioma the metabolic effects of ROS involve DNA damage responses and the Jnk pathway which modulates glucose uptake, ATP levels and hexokinase-2 and pyruvate kinase activity. Whilst damage activates antioxidative defense by activating the PPP in glioma, Jnk activation downregulates hexokinase-2 and PKM2 obstructing glycolysis, forcing reliance on oxidative phosphorylation and sensitizing glioma cells to oxidative stress (Dixit et al., 2014).

Despite increased ROS levels being of benefit to tumorigenesis, oncogenic signaling through c-Src, Ras, and Erk1/2 also amplifies the antioxidative system in order to maintain redox homeostasis (Salazar-Ramiro et al., 2016, Figure 12). By coupling redox maintenance with mitochondrial biogenesis signaling, glucose metabolism, and growth factor signaling, cancer cells are able to avoid oxidative stress (Zhang et al., 2015). Redox metabolism is coupled closely with the cell cycle, forming a reductive environment to minimize damage during DNA synthesis and anaerobic glycolysis, and an oxidative environment during mitosis driving biogenesis and proliferation (Zhang et al., 2015). This reductive state during DNA synthesis is associated with reduced functionality and ROS-dependent metastasis by boosting formation of invadopodia during oxidative phases, correlating with glioma stemness characteristics (Zhang et al., 2015). Metastasis is also affected by ROS-mediated activation of ERK and the redox-sensitive IκK/NF-κB pathway, promoting tissue digestion through metalloproteinase expression and glioma invasion (Chiu et al., 2010; Yuan et al., 2015).

## Hypoxia inducible factors and the tumor microenvironment

### Microenvironmental factors in glioma progression

Glioma progression is characterized by the appearance of extensive regions of hypoxia within the tumor microenvironment (Kucharzewska et al., 2015). The formation of a hypoxic niche is associated with tumor aggressiveness, drug resistance, and enrichment of GSCs which recapitulate the tumor following resection and therapy (Mimeault and Batra, 2013; Ye et al., 2013).

**Figure 13:**
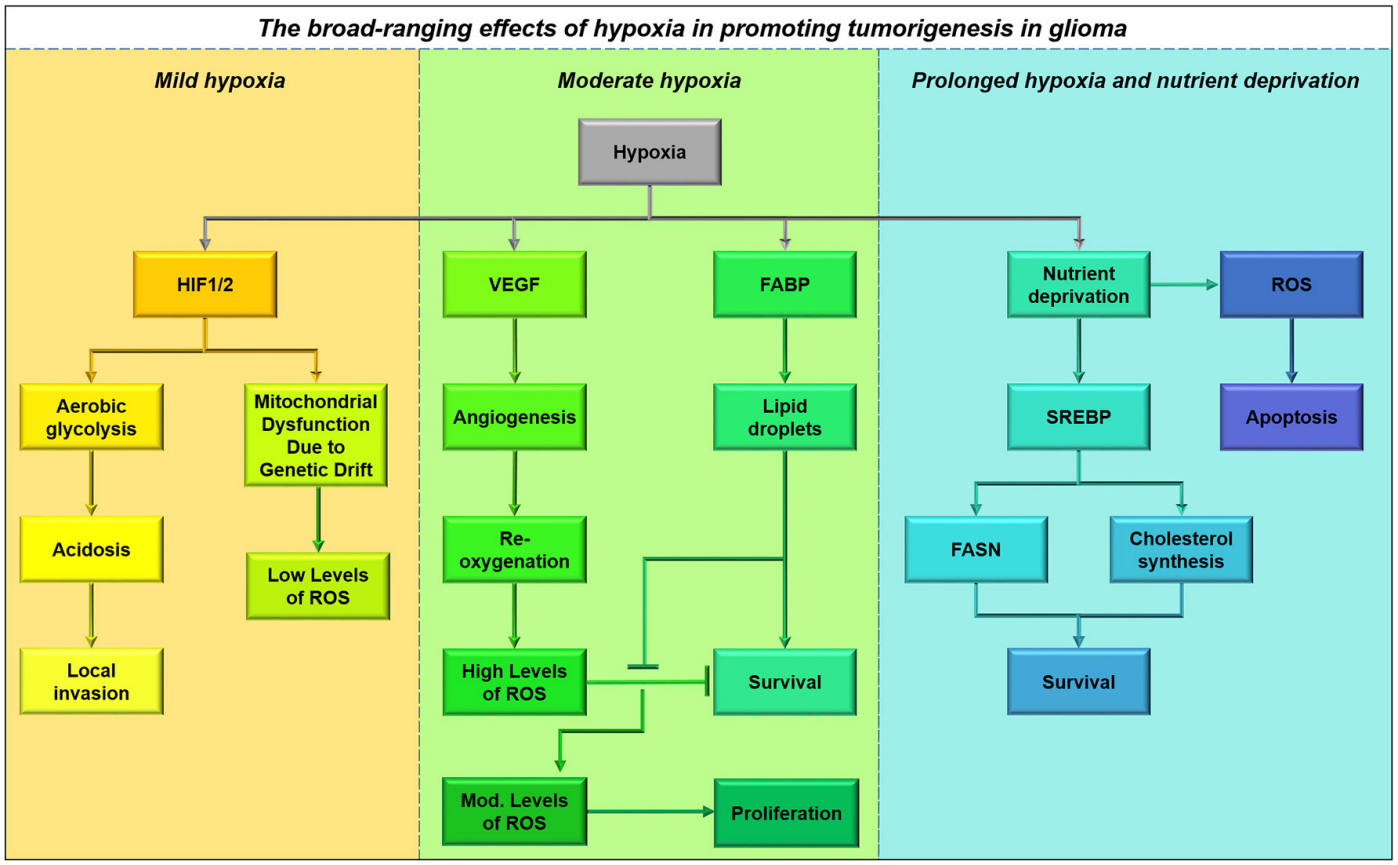
**The broad-ranging effects of hypoxia in promoting tumorigenesis in glioma, and protection from immediate ill effects of re-oxygenation**. Acute periods of hypoxia result in the up-regulation of many glycolytic genes resulting in the release of lactate and an acidic microenvironment, which is conducive for localized invasion. Hypoxia is also accompanied by mitochondrial dysfunction as a result of this metabolic adaptation, resulting in excess accumulation of ROS and apoptosis. Under conditions of moderate hypoxia, GBM cells downregulate fatty acid, and cholesterol biosynthesis pathways and primarily rely on increased fatty acid uptake to meet their nutrient requirements, through upregulation of fatty acid binding proteins particularly FABP7, resulting in the formation of lipid droplets in a time- and oxygen-dependent manner (Bensaad et al., 2014). Restricted oxygen availability and nutrient deprivation are often accompanied by poor vascularisation, causing extensive increases in apoptosis (Lewis et al., 2015). However, under these conditions some glioma cells have been shown to upregulate sterol regulatory element-binding protein (SREBP) to maintain fatty acid and cholesterol metabolism by disrupting the mevalonate pathway (Lewis et al., 2015). The accumulation of lipids in GBM helps to maintain viability upon re-oxygenation after angiogenesis, providing an alternative source for ATP production and protecting against ROS accumulation (Bensaad et al., 2014).

Hypoxia has many effects, depending upon the extent of oxygen deprivation and exposure time (Figure 13). Hypoxia causes: activation of Hypoxia Inducible Factors (HIF; the subject of this section), facilitation of adaptive metabolism (such as formation of lipid droplets), release of vascular endothelial growth factor (which influences the surrounding tissue to support neo-angiogenesis), and death (if resultant signaling and nutrient restoration does not promote survival). In fact it has been proposed that varying oxygen levels within a tumor make metabolic heterogeneity inevitable (Strickaert et al., 2016).

### Regulation of hypoxia inducible factors

**Figure 14:**
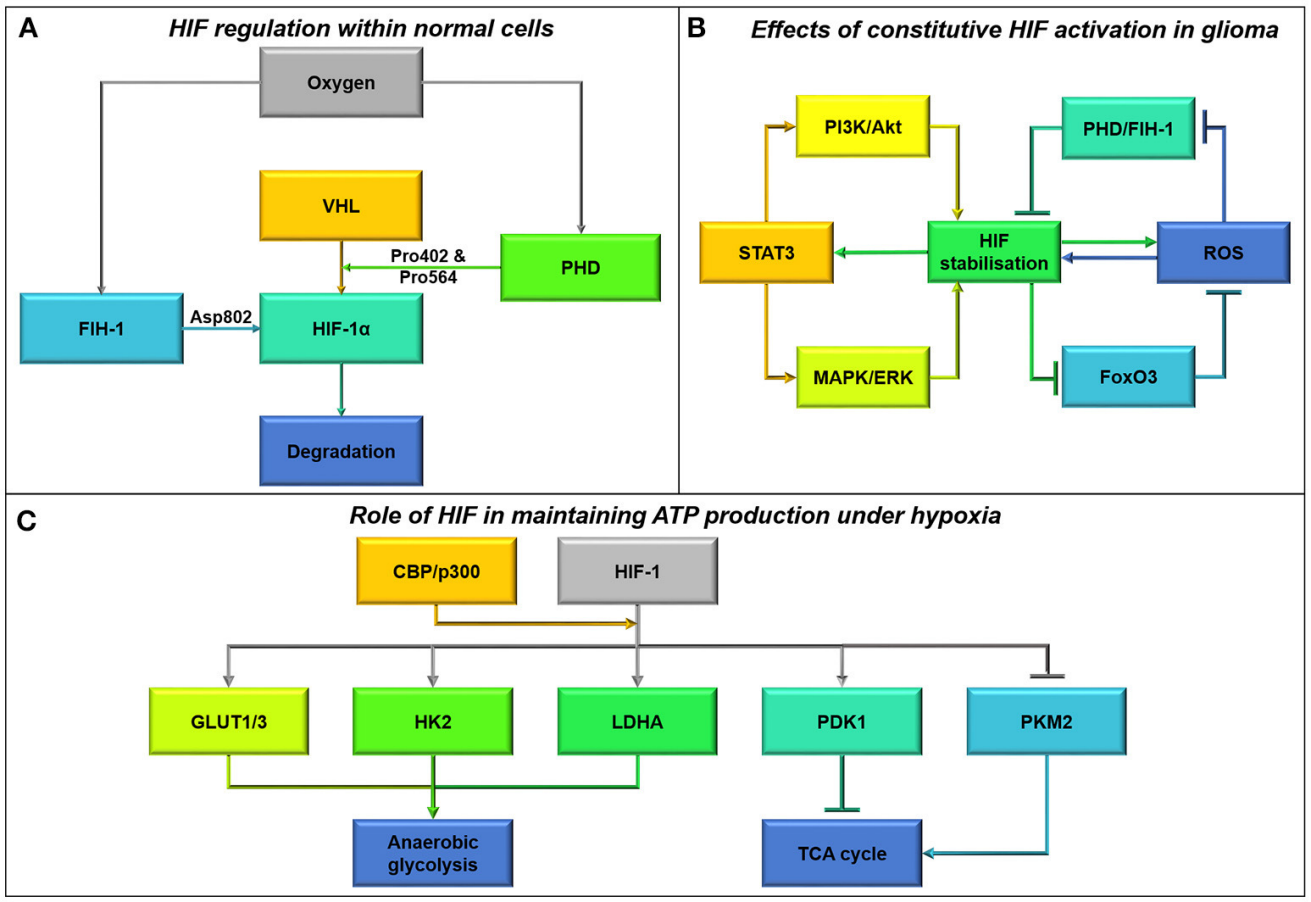
**(A)** Hypoxia-inducible factor (HIF) regulation within normal cells. There are two main avenues governing HIF-1α within normal cells, involving the factor-inhibiting hypoxia-1 (FIH-1) and Von Hippel-Lindau (VHL) proteins (Semenza, 2010). The activity of these proteins is regulated by oxygen levels, whereby FIH-1 hydroxylates HIF-1α at Asp802 blocking its transcriptional activity, whereas VHL activity is reliant on prolyl-hydroxylase (PHD) to hydroxylate Pro402 and everPro564 under oxygenated conditions, marking HIF for degradation by VHL (Semenza, 2010). However, when oxygen levels are low HIF is not marked for degradation and is therefore its transcriptional activity is unrestrained. **(B)** Effects of constitutive HIF activation in glioma. The constitutive activation of HIF under normal oxygen tension is known as pseudo-hypoxia. Oncogenic signaling in cancer cells acts to stabilize HIF under normoxia primarily through the activation of PI3K/Akt and MAPK/ERK pathways (Qiang et al., 2012; Mimeault and Batra, 2013). In turn, as a result of HIF transcription STAT3 is upregulated resulting in further activation of these oncogenic pathways (Mimeault and Batra, 2013; Qiang et al., 2012). HIF stabilization often arises in concert with ROS production, which acts to stabilize HIFs by oxidizing the catalytic iron center of PHD and FIH-1, limiting HIF degradation (Chandel et al., 2000; Semenza, 2010). Reduced FoxO3 activity as a result of oncogenic signaling and HIF transcription also acts to increase ROS production as part of a feed-forward mechanism (Ferber et al., 2012). **(C)** Role of HIF in maintaining ATP production under hypoxia. Upon stabilization the HIF-1 complex acts in concert with the CREB-binding protein (CBP)/p300 co-activator to alter the transcription of multiple genes involved in metabolism (Semenza, 2010). Through increasing the transcription of GLUT1/3, HK2, PDK1, and LDHA, HIF-1 increases glucose uptake and glycolysis whilst inhibiting the Kreb's cycle and oxidative metabolism (Semenza, 2010; Kucharzewska et al., 2015). Additionally, HIF-1 transcription also inhibits PKM2 activity to limiting flux through the Kreb's cycle and oxidative metabolism (Kim et al., 2015).

HIFs are primarily regulated by HIF prolyl-hydroxylases (PHDs) which hydroxylate Pro402 and Pro564 on HIF-1α. This allows for the recruitment of Von Hippel-Lindau ubiquitin E3 ligase which marks HIF-1α for proteasomal degradation (Semenza, 2010) (Figure 14A). As PHDs require oxygen for their activity, HIF activation is dependent on oxygen; under low-oxygen tension HIF-1α is not degraded and mediates metabolic reprogramming toward a glycolytic phenotype (Semenza, 2010). Factors inhibiting HIF-1 hydroxylate HIF-1α at Asp802, blocking the binding of p300 and CREB-binding protein and HIF-1 transcriptional activity, acting as another regulatory pathway (Semenza, 2010). In this manner HIF is able respond to alterations in the tumor microenvironment and mediate metabolic reprogramming.

Downstream of its regulation HIF-1α acts in concert with HIF-1β as a transcription factor to coordinate metabolic changes to preserve oxygen and induce angiogenesis to increase oxygen tension. HIF-1 regulates a shift from oxidative metabolism to anaerobic glycolysis when oxygen levels are depleted (Kucharzewska et al., 2015). By increasing the expression of GLUT1/3, Hexokinase-2, PDK1 and LDHA, HIF-1 induces glycolysis, whilst simultaneously upregulating PDK1 to reduce flux through the Kreb's cycle and electron transport chain, to maintain ATP levels in the absence of oxygen (Semenza, 2010; Kucharzewska et al., 2015). PDK1 also has a role in inhibiting PDH, to further amplify the cellular response to hypoxia (Kucharzewska et al., 2015).

HIFs drive metabolic reprogramming and decrease cell cycling in response to acute periods of hypoxia (Neurath et al., 2006; Kathagen et al., 2013). However, unresolved hypoxia or constitutive HIF signaling in cancer escalates glioma initiation, development, and migration (Kathagen et al., 2013).

### Constitutive HIF signaling in glioma

Hypoxia often arises concurrently with increased ROS/NOS production, which can act indirectly to stabilize HIF complexes (Chandel et al., 2000, Figure 14B). ROS/NOS oxidize the catalytic iron center of PHD and FIH and inhibit their activity to stabilize the HIF-1 complex (Semenza, 2010). Reduced FoxO phosphorylation/acetylation caused by oncogenic signaling also plays a role in stabilizing HIF-1 in ROS-dependent manner. FoxO3 transcriptional activity attenuates ROS levels, reducing HIF-1α stabilization in normal cells, however as FoxO3 activity is often downregulated due to oncogenic signaling, HIF-1 is further stabilized by increased ROS tolerance in glioma (Ferber et al., 2012). HIF signaling also results in enhanced antioxidant levels in glioma, which may act as a modulatory mechanism to maintain redox homeostasis and inhibit hypoxic death (Kucharzewska et al., 2015). Additionally, TIGAR also helps to protect cells against hypoxic death by controlling HIF-induced ROS levels (Wanka et al., 2012b).

HIF-1α expression is often increased due to oncogenic signaling. Increased HIF-1 expression requires activation of the PI3K/Akt/mTOR and MAPK/ERK pathways, and HIF-1 can further activate itself through these pathways (Qiang et al., 2012; Mimeault and Batra, 2013). Ras, a central node in controlling growth factor signaling, can increase expression of HIF-1α through augmented mTOR signaling and increased translation of HIF-1α (Semenza, 2010; Cairns et al., 2011). Activation of PDGFR in glioma also supports HIF-1 activity (Semenza, 2010). PKM2 in its tetrameric form is a target of HIF-1 metabolic remodeling to increase ATP production through glycolysis (de Wit et al., 2016). PKM2 also has a dimeric form in cancer, which has a nuclear localization sequence allowing PKM2 to act as a co-transcription factor and kinase stimulating HIF-1α expression and activity (de Wit et al., 2016).

Downstream signaling from insulin-like growth factor (IGF1) through Ras to HIF-1α upregulates mRNA encoding IGFBP2, thus autoregulating HIF by decreasing IGF1 signaling (Feldser et al., 1999; Sinha et al., 2011). IGFBP2 is often upregulated in GBM, promoting tumor development, progression, and invasion (Lin et al., 2015). Through modeling it was shown that IGFBP2 plays an integral role in sustaining HIF-1α signaling in an oxygen-independent manner due to its negative effect on IGF1 signaling (Lin et al., 2015).

### Role of HIFs in glioma progression

By upregulating glucose import and metabolism, oxygen-independent HIF regulation can induce glycolysis even in the presence of oxygen, contributing to the Warburg effect. Ras-mediated HIF-1α signaling downregulates PDH activity and mitochondrial respiration, also increasing tumorigenic potential by inducing aerobic glycolysis (Prabhu et al., 2015, Figure 14C). Hypoxic signaling in glioma also increases AMPK-mediated catabolism of proteins, in keeping with the enhanced autophagy observed in glioma and its role in conferring resistance to nutrient deprivation and therapy (Mimeault and Batra, 2013; Clark et al., 2016). By recapitulating reductive metabolism in the form of glycolysis and glutaminolysis constitutive activation of HIF-1 in cancer can contribute to cell growth (Lemaire et al., 2015).

HIF-1α has previously been reported to inhibit c-Myc activity within renal cell carcinoma to support the switch to glycolysis whilst minimizing proliferation, however in the opposite manner HIF-2α stimulated c-Myc/Max dimerization within these cells resulting in tumorigenesis (Gordan et al., 2007). Within glioma cells hypoxia upregulates serine hydroxymethyltransferase (SHMT2) due to increased HIF-1α and c-Myc activity. Increased SHMT2 activity in GBM can limit PKM2 activity, increasing PPP flux and decreasing Kreb's cycle intermediates and oxygen consumption (Kim et al., 2015). This mechanism provides selected glioma cells under hypoxia a survival advantage by increasing NADPH through mitochondrial serine degradation and protecting against ROS-induced hypoxic death (Ye et al., 2014). Increased SHMT2 is a common feature of pseudopalisading cells surrounding necrotic areas in GBM demarcating zones of angiogenesis and hypoxia for rapid re-oxygenation (Mimeault and Batra, 2013; Kim et al., 2015).

The feed-forward mechanisms supporting constitutive HIF signaling maintain the development of an invasive, metastatic and lethal phenotype in glioma (Semenza, 2010, Figure 13). Hypoxia within the glioma microenvironment results in the induction of neural stem cell markers, such as Oct3/4, Sox2, and Nestin in synergy with EMT molecules such as VEGF, which correlate with tumor aggressiveness (Qiang et al., 2012; Mimeault and Batra, 2013). By promoting stem cell maintenance and angiogenesis, hypoxia inhibits neural stem cell differentiation and promotes increased vasculature, key events in cancer progression and metastasis (Qiang et al., 2012).

Lactate production, due to HIF metabolic remodeling, also has a role in creating a favorable environment for glioma invasion (Figure 14). Lactate causes a decrease in extracellular pH forming an acidic microenvironment, which promotes the death of surrounding tissue, ECM degradation and subsequent localized migration (Kathagen et al., 2013; Mimeault and Batra, 2013). Ninety three percent of hypoxia areas show co-expression of HIF and HSP90 as a result of acidosis, further increasing the ability of GSCs to withstand nutrient deprivation and therapeutic interventions by further modulating metabolism (Filatova et al., 2016).

## Autophagy: you are what you eat

### Autophagy is dependent upon mitochondrial dynamics

In times of stress, the cancer cell may wholly or partially digest itself in a process called autophagy. In some cases, this process can lead to cell death; indeed this is a primary mechanism by which temozolomide acts to kill glioma cells (Lefranc et al., 2007). However, limited autophagy can allow survival of glioma stem cells under nutrient-deprived conditions (Sun et al., 2016), and down-regulation of these supportive autophagic processes can sensitize glioma cells to chemotherapeutics and promote apoptosis (Isakovic et al., 2017). High levels of pro-autophagy genes are associated with worse glioma patient survival, particularly in patients with the mesenchymal subtype of glioblastoma (Galavotti et al., 2013).

**Figure 15:**
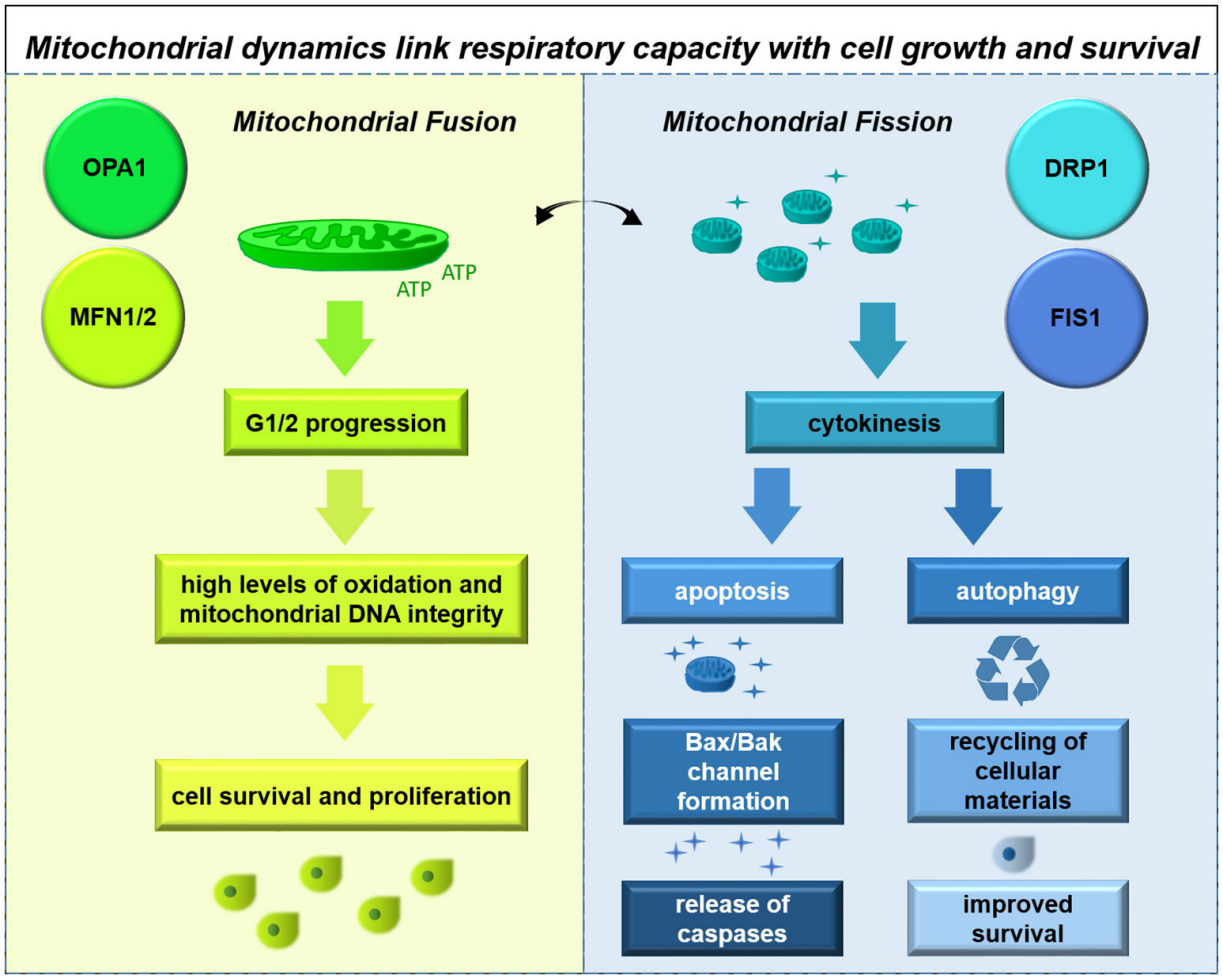
**Mitochondrial dynamics are linked with cell cycle, respiratory capacity, and cell survival**. Mitochondrial dynamics are regulated in concert with the cell cycle and coordinate metabolic activity; fusion facilitates high rates of oxidation while fission permits initiation of apoptosis or adoption of an autophagy-based survival strategy.

Autophagy is tightly controlled by mitochondrial dynamics, the process by which mitochondria undergo fusion and fission (Figure 15). Mitochondrial fusion acts as an acute compensatory mechanism to deal with cellular stress, by increasing mitochondrial DNA copy number (Chen et al., 2010), boosting expression of electron transport chain complexes, allowing rapid diffusion of metabolic intermediates (Karbowski and Youle, 2003; Twig et al., 2008) and increasing ATP production rates (Tondera et al., 2009). Fusion thereby enables a cell to manage an increased energy demand, particularly in times of stress, thus preventing the need for autophagy or apoptosis (Gomes et al., 2011; Rambold et al., 2011).

Mitochondrial fission, in contrast, reduces oxidative capacity and increases genetic drift of mitochondrial-encoded subunits of the electron transport chain (Taguchi et al., 2007). After mitochondrial membrane depolarization, any weak organelles which do not recover normal potential can undergo two fates: initiation of apoptosis or selective autophagy of the dysfunctional mitochondrion (mitophagy). The benefits of mitophagy are primarily: to remove damaged organelles which cannot contribute to cellular respiration, to prevent Bax/Bak channel formation, to reduce the quantity of caspases and other signaling factors which initiate apoptosis, and to recycle the material within the autophagosome for protein synthesis and membrane fabrication. Thus, mitophagy provides a powerful route to cellular survival, especially in times of nutrient deprivation.

### Autophagy and underlying mitochondrial dynamics influence cell cycle progression

Mitochondrial dynamics play a critical role in cancer cells, as dysregulated fusion and fission have been implicated in tumor initiation and cancer activity (Loureiro et al., 2013). Interestingly, mitochondrial dynamics act as critical cell cycle control mechanisms with highly-motile organelles arranging themselves to ensure mitochondrial homogenization and segregation during cell division (Antico Arciuch et al., 2012; Mishra and Chan, 2014). Mitochondrial fusion is required for maintaining mitochondrial membrane potential and for permitting entry into S-phase, meanwhile mitochondrial fission is required at later stages, notably to impart a mitochondrial population to each daughter cell during cytokinesis (Taguchi et al., 2007; Schieke et al., 2008; Yang et al., 2015). Although specific mechanisms linking mitochondrial fusion and fission factors to cell cycle regulatory proteins have not been characterized in glioma cells, mitochondrial dynamics do appear to be tightly tied to cell cycle progression, providing a potentially critical process linking cellular bio-energetics with growth. Indeed knockdown of the mitochondrial fission factor Drp1 in patient-derived cells reduces growth *in vitro* (Xie et al., 2015); impairments or loss of Drp1 have been associated with lower autophagic flux in other cancer cell types (Thomas and Jacobson, 2012). Intriguingly, one group has shown that stimulation of autophagy impairs invasion in glioma cell lines (Catalano et al., 2015), while another group has demonstrated that down-regulation of autophagy impairs invasion in primary human glioma cells (Galavotti et al., 2013). Further work in this area is necessary to resolve these contradictory findings.

The process of autophagy is influenced by multiple metabolic signaling pathways including AMPK, mTOR, and other factors (Westermann, 2012). Under normal conditions, PARP1 and AMPK form a complex in the nucleus of glioma cells; upon nutrient starvation this complex is disrupted, leading to nuclear export and initiation of autophagy (Rodriguez-Vargas et al., 2016). ROS signaling, often in the presence of hypoxia, activates PTEN which inhibits the PI3 kinase/mTOR pathway, thus promoting autophagic flux (Errafiy et al., 2013). Interestingly Dram1-mediated localization of the autophagy protein Sequestome 1 appears to work independently of the mTOR pathway to initiate autophagy (Galavotti et al., 2013). In addition, rapamycin-induced autophagy appears to be independent of mTOR signaling; siRNA-based knockdown of mTOR sensitizes both PTEN-wildtype and PTEN-mutant cells to rapamycin-induced cell death in a synergistic manner (Iwamaru et al., 2006), demonstrating the complexity of signaling crosstalk related to autophagy control. These processes likely work in concert to coordinate the formation of the autophagosome and guide autophagy. Autophagy remains a promising target for drug development in neuro-oncology.

## The future of glioma cell metabolism

Recent years have yielded exciting findings in the field of cancer cell metabolism, suggesting that Warburg is only a small part of the larger story. While glioma cells do partially metabolize glucose, releasing lactate into the extracellular space, other substrates are being oxidized. Yet the questions remain: What fraction of ATP is produced from glycolysis, and what fraction from oxidation? What substrates are preferentially oxidized? How easily can a glioma cell change its metabolic strategy upon exposure to hypoxia, nutrient deprivation, or acidic environment? Do different cells within the tumor have different metabolic strategies or preferred metabolic substrates? Do gliomas with different oncogenic driver mutations (e.g., in p53, NF1, or IDH) have different metabolic strategies or preferred metabolic substrates? How do glioma cells balance anabolic and catabolic needs to support growth and invasion? What is the best substrate to use as a radioligand for PET imaging in these tumors? And, of course, can these bio-energetic pathways be targeted pharmacologically to slow growth and invasion of glioma? The stage is set to enter a new era in glioma biology, by augmenting our knowledge of genetics and intracellular signaling with a more comprehensive understanding of cellular bio-energetics.

## Author contributions

MS and ES discussed the ideas and wrote the paper.

## Funding

ES was supported by Newcastle University's Institute of Neuroscience and MS performed this work as part of her degree in Newcastle University's MRes Programme in Medical and Molecular Biosciences.

### Conflict of interest statement

The authors declare that the research was conducted in the absence of any commercial or financial relationships that could be construed as a potential conflict of interest.
